# Multimodal neuroimaging unveils basal forebrain-limbic system circuit dysregulation in cognitive impairment with depression: a pathway to early diagnosis and intervention

**DOI:** 10.1016/j.tjpad.2025.100298

**Published:** 2025-07-16

**Authors:** Xiaowen Xu, Xiereniguli Anayiti, Peiying Chen, Zhongfeng Xie, Mengling Tao, Yongsheng Xiang, Mingyu Tan, Yingying Liu, Ling Yue, Shifu Xiao, Peijun Wang

**Affiliations:** a Department of Medical Imaging, Tongji Hospital, School of Medicine, Tongji University, Shanghai 200092, China; b Institute of Medical Imaging Artificial Intelligence, Tongji University School of Medicine, Shanghai 200092, China; c Department of Geriatric Psychiatry, Shanghai Mental Health Center, Shanghai Jiao Tong University School of Medicine, Shanghai, China; d Alzheimer’s Disease and Related Disorders Center, Shanghai Jiao Tong University, Shanghai, China

**Keywords:** Alzheimer’s disease, Cognitive impairment, Depression, Multimodal MRI, Basal forebrain-limbic circuit, Machine learning

## Abstract

**Background:**

Alzheimer’s disease (AD) frequently co-occurs with depressive symptoms, exacerbating both cognitive decline and clinical complexity, yet the neural substrates linking this co-occurrence remain poorly understood. We aimed to investigate the role of basal forebrain-limbic system circuit dysregulation in the interaction between cognitive impairment and depressive symptoms, identifying potential biomarkers for early diagnosis and intervention.

**Methods:**

This cross-sectional study included participants stratified into normal controls (NC), cognitive impairment without depression (CI-nD), and cognitive impairment with depression (CI-D). Multimodal MRI (structural, diffusion, functional, perfusion, iron-sensitive imaging) and plasma biomarkers were analyzed. Machine learning models classified subgroups using neuroimaging features.

**Results:**

CI-D exhibited distinct basal forebrain-limbic circuit alterations versus CI-nD and NC: (1) Elevated free-water fraction (FW) in basal forebrain subregions (Ch123/Ch4, *p* < 0.04), indicating early neuroinflammation; (2) Increased iron deposition in the anterior cingulate cortex and entorhinal cortex (*p* < 0.05); (3) Hyperperfusion and functional hyperactivity in Ch123 and amygdala; (4) Plasma neurofilamentlightchain exhibited correlated with hippocampal inflammation in CI-nD (*p* = 0.03) but linked to basal forebrain dysfunction in CI-D (*p* < 0.05). Multimodal support vector machine achieved 85 % accuracy (AUC=0.96) in distinguishing CI-D from CI-nD, with Ch123 and Ch4 as key discriminators. Pathway analysis in the CI-D group further revealed that FW-related neuroinflammation in the basal forebrain (Ch123/Ch4) indirectly contributed to cognitive impairment via structural atrophy.

**Conclusion:**

We identified a neuroinflammatory-cholinergic pathway in the basal forebrain as an early mechanism driving depression-associated cognitive decline. Multimodal imaging revealed distinct spatiotemporal patterns of circuit dysregulation, suggesting neuroinflammation and iron deposition precede structural degeneration. These findings position the basal forebrain-limbic system circuit as a therapeutic target and provide actionable biomarkers for early intervention in AD with depressive symptoms.

## 1 Background

Alzheimer’s disease (AD) is a progressive neurodegenerative disorder primarily characterized by cognitive decline and is the most common form of dementia in the elderly [1]. Mild cognitive impairment (MCI), as the prodromal stage of AD, is considered a crucial window for early detection, diagnosis, and prevention of AD [2]. Previous studies have found that depressive symptoms can persist throughout the entire course of AD. Follow-up studies show that MCI patients with depression are twice as likely to progress to dementia compared to those without depression [3]. Observational studies suggest an association between depression and a 51 % increased risk of developing dementia [4], and treatment with antidepressants in MCI patients may be associated with a delay in progression to AD by approximately three years [5]. However, the causal relationship and temporal sequence between depression and AD remain to be fully elucidated. The co-occurrence of AD and depressive symptoms significantly exacerbates the clinical complexity and neurodegenerative progression of the disease. Evidence suggests that depressive symptoms may act as an independent risk factor contributing to accelerated cognitive decline [[6], [7], [8]].

While previous studies have focused on cortical-limbic networks such as the default mode network (DMN) and monoaminergic systems like the locus coeruleus-norepinephrine (LC—NE) circuit in the pathophysiology of depression and AD, the basal forebrain-limbic circuit remains under-investigated despite its integrative role in cholinergic modulation of both cognitive and affective processes [[9], [10], [11]]. The basal forebrain uniquely projects to both the hippocampus and widespread cortical-limbic structures, serving as a convergent hub for attention, memory, and emotional regulation. Unlike monoaminergic circuits, it is particularly vulnerable to early neuroinflammatory and neurodegenerative changes in AD, which may underlie depression-specific phenotypes within the disease continuum [12]. The basal forebrain (BF) is a heterogeneous and complex structure, comprising four distinct subregions. These include the medial septal nucleus and the vertical limb of the diagonal band (Ch1–2), which provide direct cholinergic projections to the hippocampus; the intermediate portion of the diagonal band nucleus (Ch3), which specifically targets the olfactory bulb; and the nucleus basalis of Meynert (Ch4), which predominantly sends projections to the cerebral cortex, including the prefrontal, parietal, temporal, and occipital lobes, as well as the amygdala. These extensive cholinergic projections play a critical role in supporting cognitive functions, such as attention, learning, and memory [13]. Meanwhile, the limbic system includes key structures such as the hippocampus (HIP), which is central to memory consolidation and spatial navigation [14]; the amygdala (AMY), which modulates emotional processing and fear memory [15]; the entorhinal cortex (EC) serves as a critical hub for information exchange between the hippocampus and the neocortex [16]; the anterior cingulate cortex (ACC), which is implicated in conflict monitoring and emotional regulation [17]; the parahippocampal gyrus (PHG) plays a key role in the integration of spatial and episodic memory [18]. Emerging evidence suggests that dysfunctions within the basal forebrain-limbic system circuit may underlie the co-occurrence observed between AD and depressive symptoms [[19], [20], [21]]. However, the specific patterns of alterations across distinct brain regions within this circuit contributing to AD-spectrum disorders with coexisting depressive symptoms require further in-depth investigation.

Advancements in multimodal neuroimaging now enable systematic interrogation of these neural circuits. Specifically, structural MRI (sMRI) quantifies region-specific atrophy patterns, while diffusion tensor imaging (DTI) maps white matter integrity and inflammatory response through free-water fraction (FW) [22]. Resting-state functional MRI (rs-fMRI) delineates intrinsic neural circuits network dynamics via metrics including amplitude of low-frequency fluctuations (ALFF) and functional connectivity density (FCD) and so on. Arterial spin labeling (ASL) provides non-invasive quantification of cerebral blood flow (CBF), reflecting metabolic demands of neuronal activity [23]. And quantitative susceptibility mapping (QSM) provides unique sensitivity to pathological iron deposition in the basal forebrain-limbic circuit [24].

In this study, we synergistically analyze multimodal MRI features of the basal forebrain-limbic system neural circuit to systematically reveal the specific imaging patterns related to cognitive decline with/without depression, investigate the interaction between cognitive decline and depressive symptoms, and find early biomarkers for their co-occurrence. Additionally, we aim to explore the correlation between multimodal imaging features of the basal forebrain-limbic system neural circuit and AD-related biomarkers. And we will further construct machine learning-driven classification models for subtypes of cognitive impairment with or without depression.

## 2 Methods

### 2.1 Study population

This study utilized data collected from Brain Evolution Cohort (BEC) of Tongji Hospital, affiliated with Tongji University in Shanghai, between January 2020 and December 2023. The study involved normal control (NC) individuals, subjective cognitive decline (SCD), mild cognitive impairment (MCI), and Alzheimer’s disease (AD). The SCD group was defined according to the conceptual framework proposed by the Subjective Cognitive Decline Initiative (SCD-I) [25]. The diagnosis of MCI was established following the standardized criteria proposed by Jak and Bondi in 2014 [26]. AD dementia diagnoses adhered to both the neurocognitive disorder criteria outlined in the Diagnostic and Statistical Manual of Mental Disorders (5th ed.; DSM-5) and the biomarker-supported diagnostic framework for Alzheimer's disease dementia proposed by the National Institute on Aging-Alzheimer's Association (NIA-AA) workgroups [27]. The study was approved by the Ethics Committee of Tongji Hospital, affiliated with Tongji University. All participants were required to sign a written informed consent form prior to enrollment.

### 2.2 Participant grouping and criteria

These individuals underwent a comprehensive screening process, which included a medical history review, epidemiological investigation, neuropsychological assessments, and multimodal neuroimaging examinations. Demographic and clinical data were collected, including age, gender, years of education, and medical history of hypertension, diabetes, or hyperlipidemia. The neuropsychological assessments included the Mini-Mental State Examination (MMSE) [28], MoCA, Auditory Verbal Learning Test (AVLT), Shape Trail Test A (STT-A), Shape Trail Test B (STT-B), Verbal Fluency Task (VFT), Hamilton Depression Rating Scale (HAMD) [29], Hamilton Anxiety Rating Scale (HAMA) [30], and Activities of Daily Living Scale (ADL) [31].

Depending on the presence of cognitive impairment and core depressive symptoms, the participants were divided into three groups: the normal control group (NC), the cognitive impairment without depressive symptoms group (CI-nD), and the cognitive impairment with depressive symptoms group (CI-D).Specifically, the NC group was defined as having a Montreal Cognitive Assessment (MoCA) score≥26 and a Hamilton Depression Rating Scale (HAMD) score<7. CI-nD group was characterized by a MoCA score<26 and a HAMD score<7. CI-D was characterized by a MoCA score below 26 and a HAMD score of 7 and higher. A total of 709 participants were involved in this study. The MoCA cutoff of 26 was selected based on previous recommendations and validation studies, with an adjustment of +1 point for participants with less than 12 years of education [32,33]. The HAMD cutoff of <7 was used to define the absence of depressive symptoms, as established in prior research [34,35]. Clinical diagnoses were confirmed by experienced clinicians. Exclusion criteria included a history of major depressive disorder (MDD) or anxiety disorders based on DSM criteria, use of psychoactive medications, and inability to complete the study protocol or MRI scanning.

### 2.3 MRI data acquisition and calculation

For each participant, T1-weighted structural MRI, DTI, rs-fMRI, ASL, and QSM scans were conducted sequentially. All MRI data were captured using 3.0T Magnetom Verio MRI scanner (Siemens) equipped with a 32-channel head coil. Specifical scanning parameters and preprocessing steps for each modality are detailed in Table S1 and Fig. S1. Based on the map of basal forebrain subregions, we use a combination of MRI and histology, the basal forebrain was subdivided into the Medial Septal Nucleus (Ch1), Vertical Limb of the Diagonal Band (Ch2), Horizontal Limb of the Diagonal Band (Ch3), and Nucleus Basalis of Meynert (Ch4). CH123 corresponds to Ch1, Ch2, and Ch3, while CH4 refers to Ch4. The limbic system includes HIP, PHG, EC, ACC and AMY. These regions were extracted using the Julich-Brain atlas from the SPM Anatomy Toolbox Version 3.0 (https://www.fz-juelich.de/en/inm/inm-7/resources/jubrain-anatomy-toolbox) and normalized to the MNI space [36,37].

Furthermore, multimodal neuroimaging indices were systematically derived from various MRI modalities. Specifically, structural MRI (sMRI), specifically T1-weighted imaging, was used to assess gray matter volume (GMV) through voxel-based morphometry. Rs-fMRI provided functional metrics, including amplitude of low-frequency fluctuations (ALFF), regional homogeneity (ReHo), voxel-mirrored homotopic connectivity (VMHC), and intrinsic functional connectivity density (IFCD). DTI sequence was employed to calculate free-water (FW) values, while ASL sequence was utilized to quantify cerebral blood flow (CBF). Additionally, QSM sequence was applied to measure iron deposition by reconstructing susceptibility values. The specific steps for the extraction and processing of these multimodal imaging indices are described in detail in the supplementary materials.

### 2.4 Measurement of plasma biomarkers

Venous blood samples were collected from all participants using Ethylene Diamine Tetraacetic Acid (EDTA) tubes. Biomarker levels were measured using the ultra-sensitive Simoa technology on the automated Simoa HD-X platform (Quanterix, MA, US) according to the manufacturer's instructions. The plasma biomarkers analyzed in this study included amyloid-β40 (Aβ40), amyloid-β42 (Aβ42), phosphorylated tau181 (pTau181), and neurofilament light chain (NfL). These biomarkers were selected based on their established pathophysiological relevance to AD progression. Detailed rationale for biomarker selection, including their association with cerebral Aβ/Tau pathology (Aβ42/Aβ40 ratio) and neurodegeneration (NfL), along with standardized measurement protocols are comprehensively described in the supplementary materials.

### 2.5 Statistical analysis

Demographic analyses employed chi-square tests for intergroup gender comparisons, while continuous variables (age, education years, neuropsychiatric scale scores, blood biomarkers) underwent Kruskal-Wallis H tests. For inter-group difference analysis of multimodal imaging features, generalized linear models (GLM) were chosen since it allows for more flexible control of multiple covariates (such as age, gender, and years of education) and can handle more complex between-group comparisons. The GLM framework treated diagnostic groups (NC/CI-nD/CI-D) as fixed effects, with age, sex, and education as covariates, independently modeling each brain region-feature combination. To address multiplicity from 14 regions × 8 modalities, Benjamini-Hochberg false discovery rate (FDR) correction was applied at *q* < 0.05. Brain regions exhibiting significant differences (FDR-corrected *p* < 0.05) across ≥50 % of modalities were prioritized as hub discriminators. Spearman's rank-order correlations with 1000 bootstrap resamples (bias-corrected 95 % CIs) assessed blood biomarker-imaging feature relationships, focusing on the top 20 % strongest correlations for clinical interpretability. Continuous data followed normal distributions were presented as mean±SD; non-normal variables as median [Q1, Q3]. All analyses used two-tailed tests with α=0.05, implemented in SPSS 27.0.

### 2.6 Path analysis

To investigate the causal mechanisms linking neuroinflammation, structural changes in cholinergic brain regions, and cognitive function, we employed structural equation modeling (SEM) using the lavaan package in R. The details are comprehensively described in the supplementary materials.

### 2.7 Classification model construction

In order to further achieve early and accurate identification of subtypes of cognitive impairment associated with depression, we aim to construct a classification model combining multimodal imaging indicators. In this study, we employed four machine learning algorithms-support vector machine (SVM), logistic regression (LR), CatBoost, and random forest (RF)-to classify participants into three clinical subgroups: NC, CI-nD, and CI-D. The specific process of classifier training can be found in the supplementary materials.

## 3 Results

### 3.1 Demographic and clinical characteristics

**Fig. 1:**
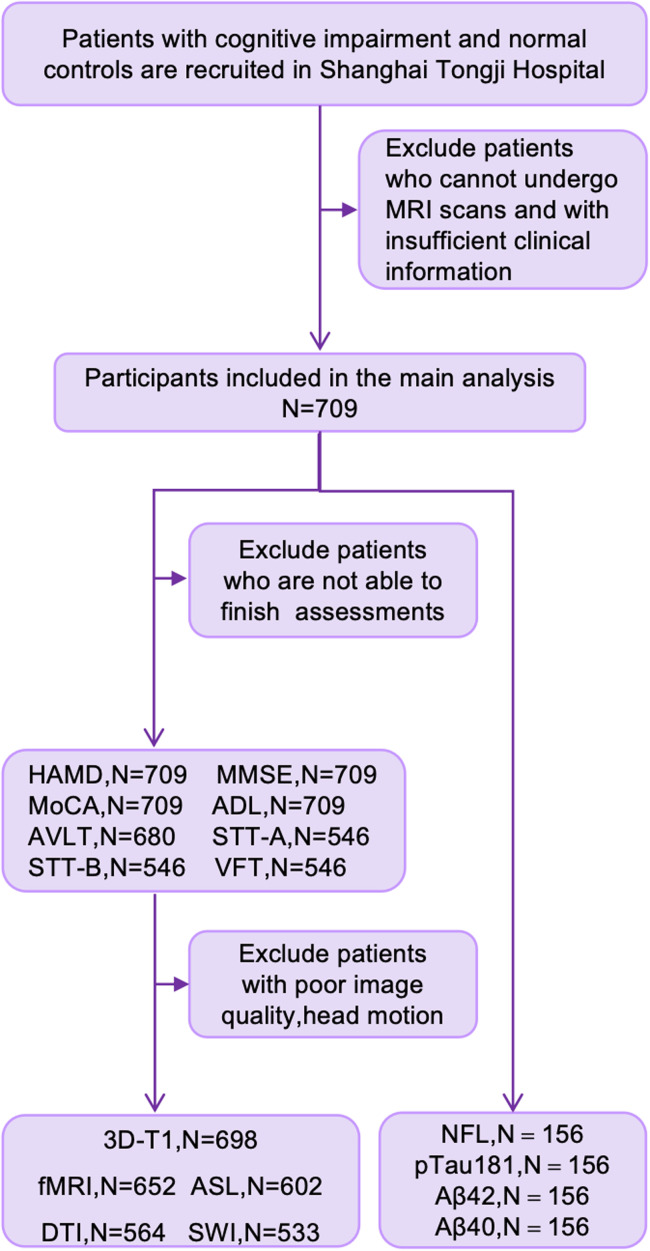
Flow chart of study design.

**Fig. 2:**
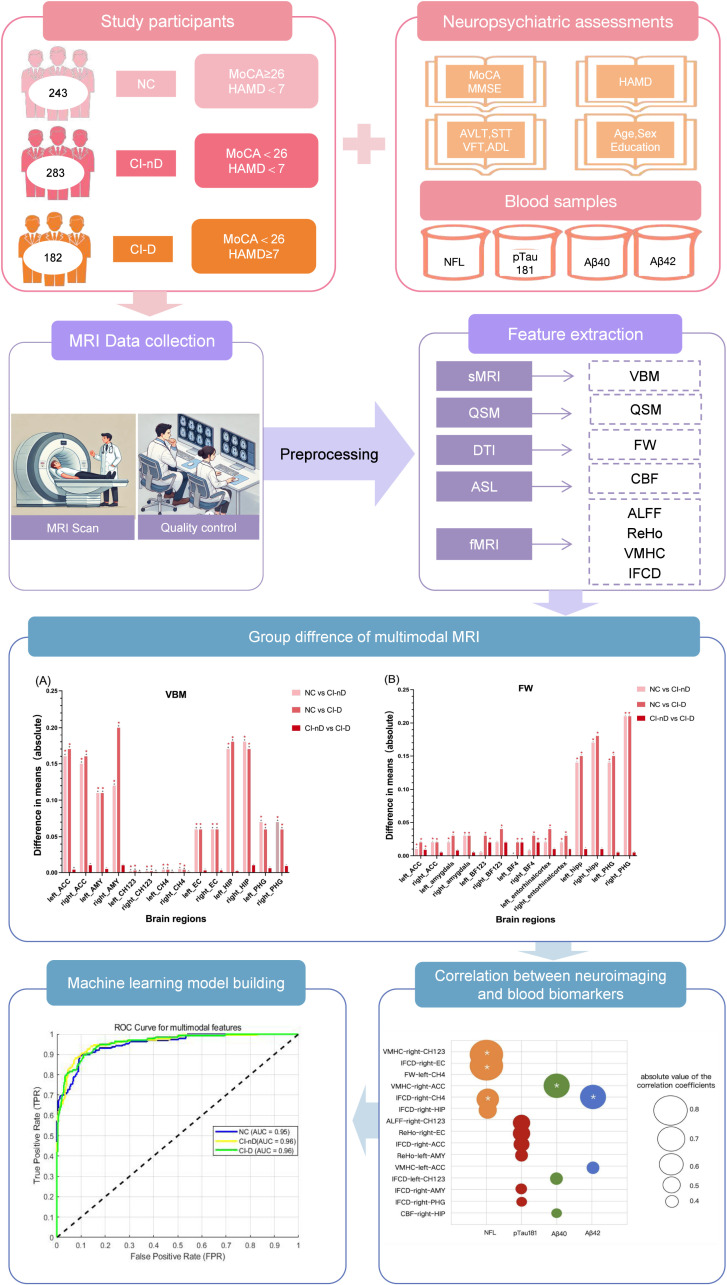
Overview of the study.

The study cohort comprised 709 participants prospectively stratified into three clinical subgroups: normal control (NC, *n* = 243), cognitive impairment without depressive symptoms (CI-nD, *n* = 284), and cognitive impairment with depressive symptoms (CI-D, *n* = 182). Participant recruitment followed the protocol detailed in Fig. 1, with comprehensive exclusion criteria ensuring sample homogeneity. Demographic analyses revealed significant intergroup differences were observed in age distribution (*p* < 0.001), with both CI-nD [72(66–77)] and CI-D [71(67–78)] groups being older than NC [69 (65–73.5)]. Educational attainment differed significantly (*p* < 0.001), with fewer years in CI-nD [9 (9–12)] and CI-D [9 (4.75–9)] compared to NC [11(9–13.5)]. Neuropsychological assessments uncovered a stepwise decline in MMSE scores across groups: NC > CI-nD > CI-D (*p* < 0.001). Similarly, MoCA, ADL, AVLT, VFT, and STT scores were significantly lower in both CI groups versus NC (all *p* < 0.001) (Table 1). Fig. 2 Fillustrated an overview of the current study design.

Note:The left half of the first column presents our study subjects and classification. A total of 709 participants were included and categorized into three groups based on the MoCA and HAMD scales: Normal control (NC), cognitive impaired non-depressive (CI-nD), and cognitive impaired depressive (CI-D). The right half displays the neuropsychological scales and blood samples collected from the patients. The second column provides an overview of the MRI data acquisition and processing, which includes scanning, quality control, preprocessing, and feature extraction. The third and fourth columns show the research findings, including bar charts depicting inter-group differences of multimodal MRI data, ROC curve analysis, and the correlation studies between blood samples and multimodal imaging data. MMSE, Mini-Mental State Examination; MoCA, Montreal Cognitive Assessment; HAMD, Hamilton Depression Scale; ADL, Activities of Daily Living; AVLT, Rey Auditory Verbal Learning Test; STT, Shape Trail Test; VFT, Verbal Fluency Test. NFL, Neurofilament Light Chain; pTau181, Phosphorylated Tau protein at threonine 181; Aβ42, Amyloid Beta 42; Aβ40, Amyloid Beta 40.VBM, voxel-based morphometry. ReHo, regional homogeneity. VMHC, voxel-mirrored homotopic connectivity. IFCD, intrinsic functional connectivity density. FW, free water. QSM, quantitative susceptibility mapping. ROC, receiver operating characteristic.

### 3.2 Multimodal MRI findings

**Fig. 3:**
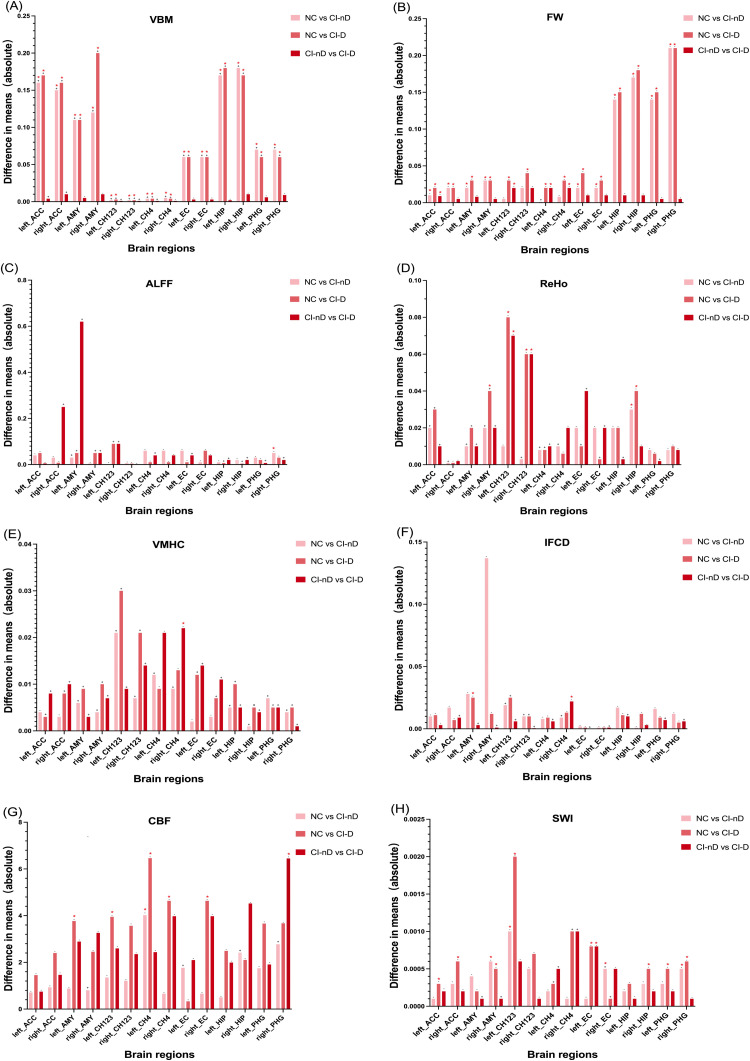
Multimodal MRI group differences in various brain regions.

sMRI-derived VBM values revealed significant gray matter volume reductions in bilateral ACC, AMY, EC, CH123, CH4, HIP, and PHG in both CI-nD and CI-D groups compared to NC (*p* < 0.001). No significant volumetric differences were observed between CI-nD and CI-D groups (Fig. 3A; Fig. S2(1)).

Note:This figure illustrates the results of group comparisons in different brain regions using a GLM model in multimodal MRI which includes VBM(A), DTI(B), fMRI(C-F), ASL(G), QSM(H). The height of the bars represents the difference in mean values between the two groups. Positive and negative signs on top of bars indicate the direction of the differences. Statistically significant differences are marked with a red asterisk.

DTI-derived FW metrics indicated significantly higher FW values in the CI-nD group compared to NC in bilateral ACC, AMY, EC, HIP, and PHG (all *p* < 0.001). In the CI-D group, elevated FW values were also observed in bilateral CH123 (*p* = 0.004, 0.01) and CH4 (*p* = 0.014, 0.001) relative to NC. Notably, depressive symptoms in the CI-D group were associated with significantly higher FW values in the left ACC, bilateral CH123, and CH4 compared to the CI-nD group (*p* < 0.040) (Fig. 3B; Figs. S2(2–4)). fMRI-extracted indicators (ALFF, ReHo, VMHC, IFCD) showed significant intergroup differences. The CI-nD group exhibited higher ALFF values in the right PHG (*p* = 0.018) and higher ReHo in the right HIP (*p* = 0.010) compared to NC. In the CI-D group, ReHo values were lower in the right AMY (*p* = 0.010) but higher in bilateral CH123 (left *p* = 0.010, right *p* = 0.030) and right HIP (*p* = 0.005) compared to NC, with bilateral CH123 showing higher ReHo in CI-D than CI-nD (left *p* = 0.028, right *p* = 0.010). VMHC values in the right CH4 were higher in the CI-D group than CI-nD (*p* = 0.032). Additionally, IFCD values in the left AMY (*p* = 0.049) and right CH4 (*p* = 0.032) were higher in the CI-D group compared to NC and CI-nD, respectively. (Fig. 3C-F; Figs. S2(5–11)).

ASL analysis revealed increased CBF in the left AMY (*p* = 0.04), CH123 (*p* = 0.031), and right CH4 (*p* = 0.049) in the CI-D group compared to NC. Both CI-nD (*p* = 0.047) and CI-D (*p* = 0.005) showed higher CBF in the left CH4 compared to NC, with a more pronounced difference in the CI-D group. Additionally, the CI-D group exhibited higher CBF in the right PHG compared to CI-nD (*p* = 0.003) (Fig. 3G; Figs. S2(12–14)).

QSM analysis revealed significant differences in both CI groups compared to NC. The CI-nD group showed higher QSM values in the right AMY and lower QSM values right EC compared to the NC group (*p* = 0.022, 0.038). In the CI-D group, QSM values were elevated in the bilateral ACC, right HIP, and left PHG (*p* < 0.05) compared to the NC group. Both CI groups exhibited higher QSM values in the left CH123 and right PHG, with more pronounced differences in CI-D. Additionally, the CI-D group had significantly higher QSM values in the left EC compared to both CI-nD (*p* = 0.033) and NC (*p* = 0.028) (Fig. 3H; Figs. S2(15–17)).

### 3.3 Summary of key multimodal imaging differences

**Fig. 4:**
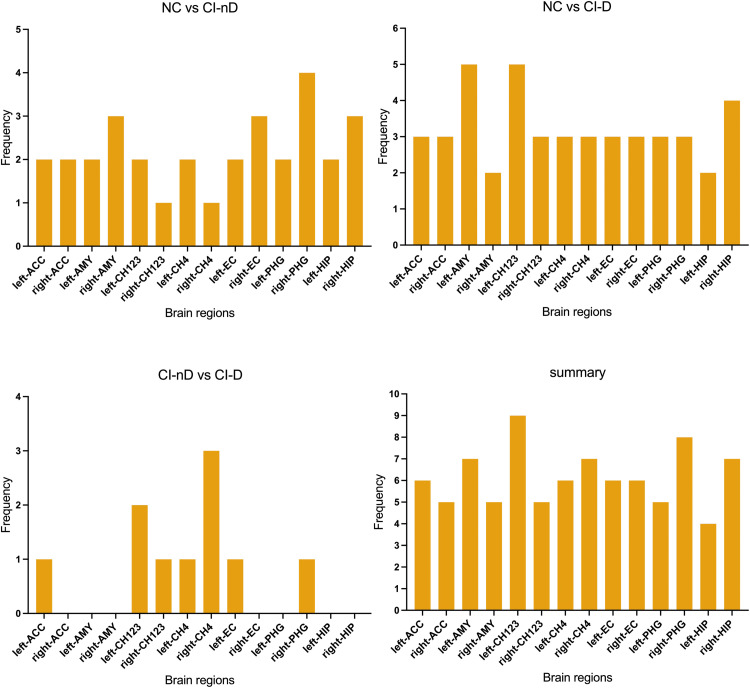
Frequency of significant brain regions differences across groups.

**Fig. 5:**
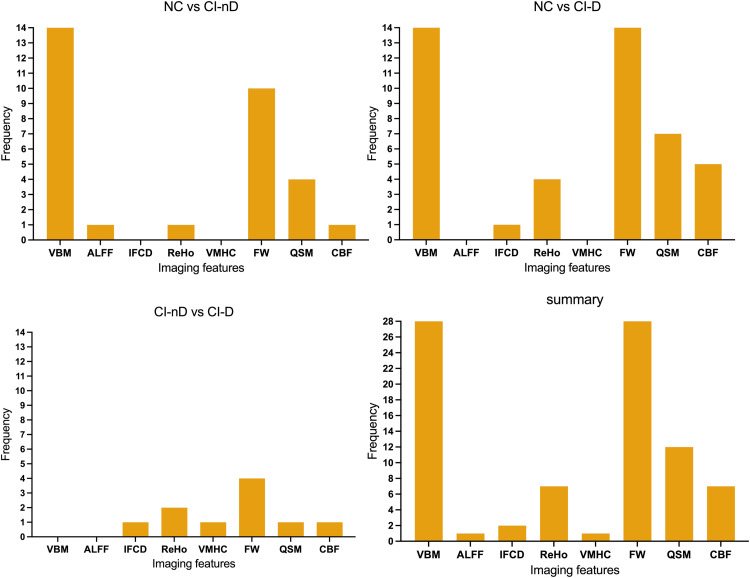
Frequency of significant imaging marker differences across groups.

Table S2 summarized the significant group differences across various brain regions. Specific patterns of multimodal imaging differences in neural circuits were shown in Figs. 4–5. From the ROI brain regions with significant differences in neural circuits between the three groups, our results found that the right AMY, right EC, right PHG, and right HIP exhibited the highest differential frequencies between NC and CI-nD groups. In contrast, comparisons between NC and CI-D predominantly implicated the left AMY, left CH123, and right HIP. When comparing CI-nD and CI-D, key divergent regions included left CH123 and right CH4. Consistent discriminators across all comparisons included left CH123, right PHG, right HIP, and left AMY.

Additionally, an analysis of the multimodal imaging markers reflecting significant neural circuit differences across the three groups revealed that VBM and FW exhibited the greatest divergence between NC and CI-nD groups. In contrast, differences between NC and CI-D groups were characterized by alterations in VBM, FW, and QSM, while comparisons between CI-nD and CI-D groups primarily highlighted FW and ReHo. Notably, VBM, FW, and QSM collectively demonstrated substantial discriminative capacity as multimodal biomarkers.

**Table 1:** Characteristics of the study population.

Characteristics	NC	CI-nD	CI-D	*p* value	*p* value		
					NC vs CI-nD	NC vs CI-D	CI-nD vs CI-D
N	243	284	182				
Female	138(56.7 %)	63(22.2 %)	118(64.8 %)	0.187^a^	0.30	0.23	0.09
Age(years)	69(65–73.5)	72(66–77)	71(67–78)	<0.001^b^	0.01*	0.002*	1
Education (years)	11(9–13.5)	9(9–12)	9(4.75–9)	<0.001^b^	<0.001*	<0.001*	0.322
MMSE	28(26–29)	24(20–26)	21(16.75–24)	<0.001^b^	<0.001*	<0.001*	0.028*
MoCA	23(21–25.5)	17(12–20)	14(10–18)	<0.001^b^	<0.001*	<0.001*	1
HAMD	1(0–4)	2(0–3.25)	10(8–13)	<0.001^b^	1	<0.001*	<0.001*
ADL	14(14–14)	14(14–16)	16(14–23)	<0.001^b^	<0.001*	<0.001*	<0.001*
N	227	278	175				
AVLT Learning	17(14–20)	12(10–16)	11.5(8–15.2)	<0.001 ^b^	<0.001*	<0.001*	1
AVLT Delayed recall	5(4–7)	3(0–5)	3(0–4)	<0.001^b^	<0.001*	<0.001*	1
AVLT Recognition	5(3–7)	1(0–4)	1(0–4)	<0.001^b^	<0.001*	<0.001*	1
N	181	215	150				
STT-A	70(52–84)	81(64–128)	85.5(63 −104)	<0.001^b^	<0.001*	<0.001*	1
STT-B	164(137–219)	215.5(155–268)	191(145–221)	<0.001^b^	<0.001*	<0.001*	1
VFT	14(11.8–16)	10(6.3–12.8)	10.5(9–12)	<0.001^b^	<0.001*	<0.001*	1

Notes: Raw data were presented as median (first quartile-third quartile) or number (percentage,%).

⁎ Indicates a significant statistical difference between groups, *p*<0.05, the significance level has been adjusted using the Bonferroni correction method. ^a^ The *p* value was obtained by Chi-square test. ^b^ The *p* value was obtained by Kruskal-Wallis test.

### 3.4 Correlation analysis between multimodal imaging features and biomarkers

We conducted a comprehensive analysis to examine the relationship between neuroimaging alterations and core AD's biomarkers, specifically Aβ42 and pTau181. Blood biomarker analysis revealed that pTau181 levels were significantly elevated in the CI-nD group compared to NC (*p* = 0.035). In contrast, both the CI-nD and CI-D groups exhibited higher levels of Aβ42 (*p* = 0.017) and Aβ40 (*p* = 0.003) relative to the NC. However, no significant differences were observed between the CI-nD and CI-D groups for these biomarkers. Furthermore, levels of NFL did not differ significantly across the three groups (Table 2).

**Table 2:** Intergroup differences in blood biomarkers.

	NC	CI-nD	CI-D	*p* value		
				NC Vs CI-nD	NC Vs CI-D	CI-nD Vs CI-D
N	88	52	16			
NFL	16.23(13–21.1)	18.39(13.5–24.1)	18.7(15–24.7)	0.50	0.23	0.29
pTau181	1.85(1.4–2.4)	2.36(1.7–3.7)	2.39(1.8–3.7)	0.035*	0.098	1
Aβ42	5.89(4.8–9.3)	8.3(6.1–10.3)	9.56(5.6–12)	0.017*	0.012*	1
Aβ40	104(88.3–151)	157(101–207.4)	190(108–244)	0.003*	0.014*	1

**Fig. 6:**
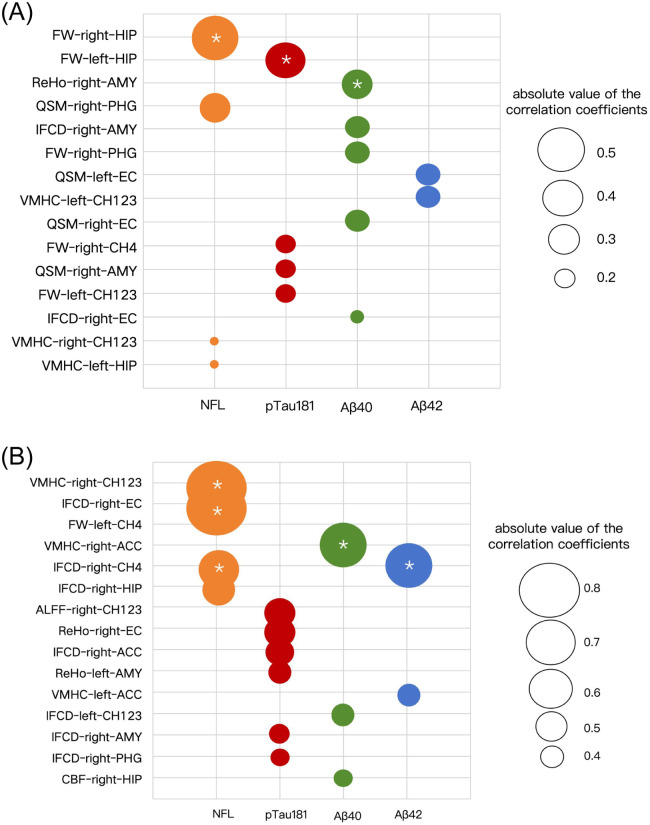
Correlation between blood biomarkers and brain imaging features in CI-nD and CI-D group.

Note: This figure presents a bubble chart showing the Spearman's correlation between blood biomarkers and brain regions in the CI-nD group (A) and CI-D group(B). Asterisks (*) indicate the brain regions with the strongest correlations with blood biomarkers in each group. Bubble size represents the strength of the correlation between imaging features and blood biomarkers.

Spearman’s correlation analysis between blood biomarkers and imaging features revealed that in the CI-nD group, the HIP and AMY constituted the highest-ranking 20 % of brain regions associated with blood biomarkers, whereas in the CI-D group, the top 20 % of biomarker-correlated regions included the CH123 and CH4 subregions, EC, ACC, and right CH4 (Fig. 6, Table S3–4).

### 3.5 Investigating the mediating role of basal forebrain atrophy in neuroinflammation-related cognitive decline

Pathway analysis was performed in the CI-D group to assess the indirect effects of FW changes in the basal forebrain (CH123 and CH4 regions) on cognitive function via structural atrophy quantified by VBM (Fig. S3). For the CH123 region, higher FW was significantly associated with reduced gray matter volume (β = –0.480, *p* < 0.001), which in turn predicted lower MoCA scores (β = 0.224, *p* = 0.012). The indirect effect (FW → VBM → MoCA) was found to be significant (β_indirect = –0.108, *p* = 0.020), accounting for 47 % of the total effect. In the CH4 region, elevated FW values were associated with a reduction in gray matter volume (β = –0.256, *p* < 0.005). Although the association between gray matter volume and MoCA scores did not reach statistical significance (β = 0.224, *p* = 0.09), the indirect effect of FW on cognition via VBM (FW → VBM → MoCA) was significant (β_indirect = –0.071, *p* = 0.030).

### 3.6 Classification of NC, CI-nD, and CI-D groups

**Fig. 7:**
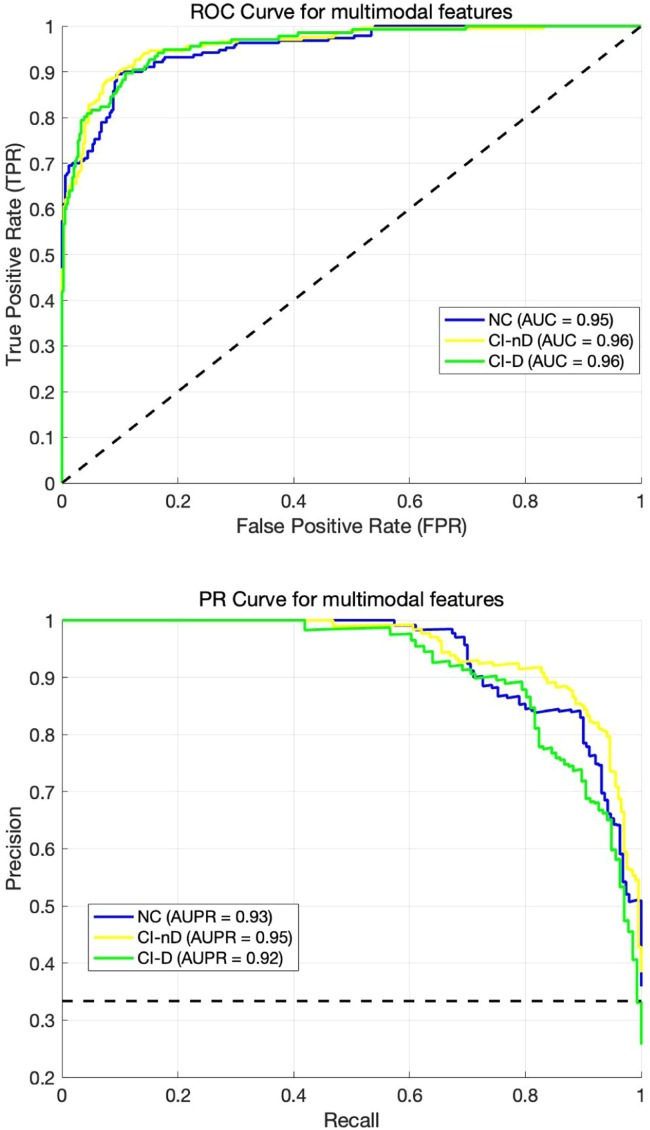
The ROC and PR curves for multimodal SVM model.

In the present study, a total of 529 subjects from the BEC cohort, who met rigorous criteria for data completeness and homogeneity, were included in the construction of classification models. A comparative analysis of four machine learning algorithms for classifying NC, CI-nD, and CI-D groups demonstrated that the SVM-based multimodal fusion model outperformed single-modality approaches (Table 3). The multimodal SVM achieved optimal accuracy (0.85), with an accuracy of 0.85, sensitivity of 0.90, specificity of 0.88, and an AUC of 0.95 for NC vs. CI-nD classification. For NC vs. CI-D, the model achieved a sensitivity of 0.82, specificity of 0.93, and an AUC of 0.96, while for CI-nD vs. CI-D differentiation, it demonstrated a sensitivity of 0.81, specificity of 0.95, and an AUC of 0.96. The ROC and PR curves for this superior model were presented in Fig. 7. Detailed performance metrics for all models were provided in Table S5–8.

**Table 3:** Comparative performance of optimal single-modality and multimodal models.

Feature	Model	Accuracy	Sensitivity	Specificity	AUC
ALFF	CatBoost	0.70	1.00/0.75/0.20	1.00/0.67/0.89	1.00/0.79/0.79
ReHo	SVM	0.74	1.00/0.78/0.16	1.00/0.77/0.22	1.00/0.77/0.74
IFCD	RF	0.72	1.00/0.88/0.13	1.00/0.69/0.67	1.00/0.79/0.77
VMHC	RF	0.75	1.00/0.75/0.37	1.00/0.59/0.45	1.00/0.86/0.84
GMV	RF	0.75	1.00/0.88/0.23	1.00/0.67/0.79	1.00/0.83/0.81
FW	RF	0.75	1.00/0.88/0.27	1.00/0.78/0.69	1.00/0.83/0.82
QSM	RF	0.74	1.00/0.81/0.27	1.00/0.68/0.67	1.00/0.80/0.76
CBF	CatBoost	0.72	1.00/0.75/0.27	1.00/0.73/0.64	1.00/0.77/0.76
Multimodal fusion	SVM	0.85	0.90/0.82/0.81	0.88/0.93/0.95	0.95/0.96/0.96

**Note:** Sensitivity, specificity, and AUC values are listed in the order of NC, CI-nD, and CI-D groups, separated by slashes ("/").

## 4 Discussion

This multimodal neuroimaging study delineates a distinct neurobiological pathway of basal forebrain-limbic circuit dysregulation in mediating the co-occurrence of cognitive impairment and depressive symptoms. By integrating structural, functional, and metabolic imaging biomarkers, we demonstrate that depressive symptoms with cognitive impairment are associated with distinct spatiotemporal patterns of neuroinflammation (elevated free-water fraction), iron dyshomeostasis (QSM), functional network reorganization (ALFF/ReHo/VMHC/IFCD) and perfusion (CBF).

A key innovation of this study is the identification of early neuroinflammatory-cholinergic dysfunction in the basal forebrain, particularly in the Ch123 and Ch4 subregions. The pronounced FW elevations observed in regions such as Ch123 and Ch4 in the CI-D group may suggest that neuroinflammation may precede overt structural degeneration [38]. These findings align with postmortem evidence of microglial hyperactivation in the basal forebrain of AD-depression cohorts, and extend recent studies linking FW elevations to resistance to antidepressant treatments, underscoring the pivotal role of neuroinflammation in exacerbating both affective and cognitive dysfunctions [[39], [40], [41]]. However, it is important to note that this inference is hypothetical and supported only by indirect evidence. Future longitudinal studies are needed to further explore the relationship between the potential reversibility of functional connectivity changes and irreversible structural loss.

Moreover, our study identifies a novel mechanistic pathway through which neuroinflammation, and cholinergic dysfunction contribute to the propagation of Aβ and Tau pathology. Elevated ReHo, IFCD, and CBF may reflect maladaptive compensation or excitotoxicity, but this interpretation is speculative. These changes could also indicate vascular dysregulation or regional disinhibition, as observed in AD and late-life depression. Notably, similar functional alterations have been reported in studies of late-life depression and AD animal models, lending support to these interpretations [42,43]. These findings are consistent with preclinical models of AD and depression, where hyperactivity in emotional regulation networks emerges as an adaptive response to neuroinflammatory stress. This hyperactivity may contribute to the accelerated deterioration of cognitive-emotional networks, thus amplifying the progression of both emotional and cognitive dysfunctions [[44], [45], [46], [47]]. Increasing evidence suggests that neuropsychiatric disorders are better conceptualized as network-level dysfunctions rather than isolated regional abnormalities. Functional connectivity network mapping studies in schizophrenia and suicidality have identified consistent disease-specific networks involving the default mode, salience, and frontoparietal systems, offering a unifying framework for heterogeneous imaging findings and guiding neuromodulation targets [[51], [52], [53], [54]]. Similarly, recent multimodal MRI research in AD has revealed progressive gray and white matter changes linked to microbiota-metabolite alterations and cognitive decline, highlighting the relevance of systems-level pathology [55]. These insights support the future application of network-informed stimulation strategies. Our results provide novel insight into how depressive symptoms may not only exacerbate but also accelerate the cognitive decline observed in AD patients. This suggests that depression may play an active, rather than a secondary, role in the progression of AD continuum.

Additionally, the integration of iron-sensitive imaging through QSM further reveals the critical role of iron dyshomeostasis in the cognitive impairment combined with depressive symptoms. The elevated QSM values in regions such as the basal forebrain and hippocampus in the CI-D group, particularly in areas associated with emotional and cognitive processing, suggest that iron accumulation contributes to neurodegeneration by increasing oxidative stress and disrupting neuronal function. These findings have important clinical implications, suggesting that targeting iron dysregulation or neuroinflammation may represent potential therapeutic avenues for AD patients with depressive symptoms. Previous studies also suggest that iron-targeted therapies, such as iron chelators (e.g., deferoxamine [DFO], deferiprone [DFP], and deferasirox [DFX]), have shown promise in preclinical and early clinical studies by reducing iron accumulation, oxidative stress, Aβ aggregation, and tau phosphorylation [[48], [49], [50]].

Our machine learning models significantly advance the field by identifying specific biomarkers for depression subtyping within the basal forebrain, with Ch123 and Ch4 emerging as key discriminative regions. These findings are pivotal, as they provide a more nuanced understanding of the brain regions involved in the co-occurrence of cognitive impairment and depressive symptoms, extending beyond the conventional perspective of depression as a secondary emotional response to cognitive decline. The high classification accuracy achieved by our models further underscores the potential of multimodal imaging in enhancing diagnostic precision and facilitating the early identification of patients at risk for depressive symptoms during the early stages of AD. By focusing on these specific basal forebrain subregions, our results offer a promising foundation for the development of targeted neuromodulatory interventions, such as deep brain stimulation (DBS) or cholinergic therapies, aimed at mitigating the neurodegenerative processes that drive both depression and cognitive decline in AD. Moreover, these findings highlight CH123 and CH4 as critical biomarkers to guide both pharmacological and non-invasive interventions. These markers could prioritize candidates for cholinesterase inhibitors or anti-inflammatory therapies targeting cholinergic dysfunction and neuroinflammation in the basal forebrain [51]. Additionally, non-invasive techniques like transcranial magnetic stimulation (TMS) and transcranial direct current stimulation (tDCS) may leverage CH123/CH4 dysfunction to target regions involved in cognitive-emotional regulation [52,53]. Integrating these biomarkers into clinical practice could thus enhance personalized strategies for managing depression in AD.

Although plasma biomarkers like pTau181 and Aβ42/40 did not distinguish CI-nD from CI-D, their region-specific associations revealed subtype-dependent divergence. In CI-nD, elevated Tau and Aβ correlated strongly with hippocampal and amygdala degeneration, consistent with the limbic-predominant AD trajectory [[54], [55], [56]]. In contrast, CI-D showed identical plasma biomarkers but distinct linkages to functional abnormalities in the basal forebrain (CH123/CH4), EC and ACC, suggesting cholinergic-limbic circuit dysregulation as a novel pathway for depressive symptoms. The combination of plasma and imaging biomarkers holds great promise for improving the differentiation of depressive phenotypes in AD. While plasma biomarkers provide useful information about systemic protein accumulation, imaging biomarkers allow for a more nuanced understanding of the spatial and functional changes occurring in the brain. As our study demonstrates, integrating both modalities could improve diagnostic classification, providing more accurate insights into the complex interplay between depression and AD's pathology.

Our results demonstrated that neuroinflammatory and iron-related abnormalities within the basal forebrain-limbic circuit-particularly in Ch123, Ch4, and the entorhinal cortex-were spatially associated with alterations in plasma Aβ and pTau levels. These findings are consistent with growing evidence that early cholinergic dysfunction may promote tau propagation and hinder amyloid clearance via glymphatic and neuroimmune mechanisms [57,58]. Concurrent increases in functional activity and cerebral perfusion in these regions may reflect maladaptive compensatory processes or stress-induced vulnerability, which in turn may accelerate pathological protein accumulation [59,60]. Taken together, these results support a spatiotemporal model in which early neuroinflammatory-cholinergic disruption amplifies Aβ and tau toxicity within emotion–memory circuits, contributing to both cognitive and affective decline. This perspective advances beyond a purely molecular framework, highlighting how circuit-level vulnerability-driven by inflammation, iron dyshomeostasis, and connectivity alterations-may underlie the clinical and biological heterogeneity observed across the AD spectrum.

This study leveraged an extensive and well-characterized cohort, utilized a robust multimodal neuroimaging metrics, and integrated plasma biomarkers and machine learning models to derive clinically meaningful subgroup differentiation. This comprehensive approach not only deepens our mechanistic understanding of the interplay between cognitive impairment and depressive symptoms but also strengthens the potential for future biomarker-guided precision interventions in AD. However, there were still some limitations that should be addressed in future research. First, the cross-sectional design precludes causal inference regarding the temporal relationship between depressive symptoms and cognitive decline. A larger sample size, including blood samples, neuroimaging data, and longitudinal follow-up, would improve the power to detect subtle effects and track the progression of cognitive impairment and depressive symptoms over time. Second, although our multimodal imaging framework provides a comprehensive view of basal forebrain-limbic circuit alterations, certain relevant brain regions, such as DMN, were not included in the present analysis. Finally, the generalizability of findings may be limited due to the single-center design and demographic characteristics of our cohort. The robustness of our findings requires confirmation in larger, multi-center cohorts.

In conclusion, we offer a framework for understanding the interaction between cognitive impairment with depressive symptoms. Our findings highlight the potential for early intervention by integrating multimodal neuroimaging biomarkers in AD patients with depressive symptoms. Biomarker-guided therapies, such as neuromodulation or anti-inflammatory treatments targeting the basal forebrain, may disrupt the pathological loop between depression and AD, offering a promising strategy to slow disease progression.

## Consent statement

The study was approved by the Ethics Committee of Tongji Hospital affiliated with Tongji University (ChiCTR2000030614). Written informed consent was obtained from all participants. All BEC participants provided written informed consent in compliance with the Declaration of Helsinki.

## CRediT authorship contribution statement

**Xiaowen Xu:** Writing – review & editing, Validation, Supervision, Conceptualization. **Xiereniguli Anayiti:** Writing – original draft, Visualization, Software, Methodology, Investigation, Formal analysis, Data curation. **Peiying Chen:** Formal analysis, Data curation. **Zhongfeng Xie:** Software, Resources, Project administration. **Mengling Tao:** Visualization, Validation, Resources. **Yongsheng Xiang:** Software, Methodology. **Mingyu Tan:** Project administration, Methodology, Investigation. **Yingying Liu:** Methodology, Data curation. **Ling Yue:** Validation, Supervision, Funding acquisition. **Shifu Xiao:** Validation, Supervision, Funding acquisition. **Peijun Wang:** Validation, Supervision, Resources, Project administration, Investigation, Funding acquisition, Conceptualization.

## Declaration of competing interest

The authors declared no conflict of interests.

## Appendix Supplementary materials

Image, application 1
