# Supplementary material for: Multimodal neuroimaging unveils basal forebrain-limbic system circuit dysregulation in cognitive impairment with depression: a pathway to early diagnosis and intervention
Source: J Prev Alzheimers Dis. 2025 Jul 16;12(8):100298. doi: 10.1016/j.tjpad.2025.100298 (PMC12413736; doi:10.1016/j.tjpad.2025.100298)
Supplement: Supplementary file 1 [file mmc1.docx]

**1.MRI data acquisition and quality control**

To ensure the quality of fMRI data and minimize the impact of head motion on subsequent analyses, a series of preprocessing steps were applied. First, the framewise displacement (FD) of head motion was calculated for all participants, and only those with an average FD of less than 0.5 mm were included in the study. During preprocessing, head motion correction was performed by aligning the functional images to the mean functional image used as a reference. Additionally, head motion-related signals were regressed out to further reduce their influence on the results. These measures ensured that head motion artifacts were adequately controlled, thereby enhancing the reliability of the analyses.

**2.Feature selection of multimodal MRI**

The selection of imaging features in this study is based on their extensive application and biological relevance in research on cognitive decline and emotional disorders. Specifically, T1WI, as a standardized structural imaging modality, provides high-resolution anatomical information, enabling the evaluation of gray matter volume (GMV) changes. GMV alterations have been widely implicated in regions critical to cognition and emotional regulation[1].

Rs-fMRI features, including amplitude of low-frequency fluctuation (ALFF), regional homogeneity (ReHo), voxel-mirrored homotopic connectivity (VMHC), and intrinsic functional connectivity density (IFCD), capture various aspects of brain functional network activity. ALFF reflects the intensity of spontaneous neuronal activity via frequency-domain analysis, providing direct insight into regional metabolic and functional states. ReHo measures the synchronization of time-series signals among neighboring voxels, indicating the coherence of local functional networks[2]. VMHC quantifies functional connectivity symmetry between homologous regions in the two hemispheres, offering insight into interhemispheric communication[3]. IFCD evaluates the density of functional connections across the entire brain, reflecting global functional network integration[4]. ReHo evaluates the similarity of the time series of a given voxel with its adjacent voxels, providing insights into the coherence of local functional networks. This metric is particularly useful for identifying regional abnormalities in brain function associated with cognitive and emotional disorders[5].

DTI, specifically free-water (FW) analysis, is a sensitive measure of microstructural integrity in white matter. By separating tissue and free-water compartments, FW analysis enables the detection of pathological changes such as inflammation, edema, or neurodegeneration in white matter tracts. These changes are frequently associated with depressive disorders and cognitive impairments, making FW a robust indicator of white matter pathology[6].

QSM, an advanced MRI technique, measures magnetic susceptibility in brain tissue, offering unique insights into microstructural pathologies such as iron deposition. Iron dysregulation, particularly in subcortical structures like the thalamus, pallidum, and putamen, is strongly implicated in neurodegenerative and emotional disorders, making QSM a critical tool for investigating these conditions[7].

Lastly, ASL provides non-invasive quantification of cerebral blood flow (CBF), a key hemodynamic measure that directly reflects neuronal metabolic and functional activity. CBF alterations have been extensively reported in cognitive decline and emotional disorders, making it a valuable feature for investigating regional and global changes in brain perfusion[8].

**3.Feature calculation of multimodal MRI**

**3.1 T1-weighted structural imaging**

We utilized voxel-based morphometry (VBM) to extract volumetric data from 14 target brain regions, including the left and right ACC, AMY, CH123 andCH4, EC, HIP, PHG. Initially, high-resolution T1-weighted images were acquired using standard structural magnetic resonance imaging (MRI) scans. MRI data were processed using CAT12 (http://www.neuro.uni-jena.de/software/), a toolbox integrated into SPM12 (http://www.fil.ion.ucl.ac.uk/spm/software/spm12). Preprocessing followed the default CAT12 pipeline, which included: (i) segmentation of gray matter (GM), white matter (WM), and cerebrospinal fluid (CSF); (ii) normalization to the standard MNI space with modulation to account for volumetric changes; and (iii) spatial smoothing using an 8-mm full-width at half-maximum (FWHM) Gaussian kernel. Total intracranial volume (TIV) was also computed.

Standard anatomical templates or regions of interest (ROIs) based on previous studies were used for the precise segmentation of the target brain regions. The gray matter volume within each ROI was calculated from the preprocessed data. For each brain region, the total volume was derived by counting the number of voxels within the normalized structural images and multiplying by the voxel size. To account for individual differences in brain size, the extracted regional volumes were standardized, typically by dividing each region's volume by the total brain volume or intracranial volume (ICV), resulting in relative volume values. Subsequently, quality control measures were applied to the extracted volume data to eliminate noise or outliers, and statistical methods were employed to assess the normality and uniformity of the data, ensuring the reliability of the features. Finally, the standardized volumetric data of the brain regions were compiled into a feature matrix for subsequent statistical analysis[9].

**3.2 rs-fMRI**

In this study, we analyzed several fMRI-based metrics, with a focus on four key indicators: ALFF, ReHo, VMHC, and IFCD. First, ALFF was calculated by performing a Fast Fourier Transform (FFT) on the time-series data of each voxel, converting the data from the time domain to the frequency domain. The power spectrum density within a specific low-frequency range (typically 0.01-0.08 Hz) was then calculated, followed by taking the square root of the power within this range to obtain the amplitude value. Higher ALFF values indicate greater neural activity intensity in the region. ReHo was computed using Kendall's W coefficient to assess the consistency of BOLD（Blood Oxygenation Level-Dependent） signal time-series between each voxel and its 27 neighboring voxels. This coefficient reflects the synchronization of neural activity within a brain region, with higher ReHo values indicating stronger local functional coherence. VMHC was used to measure functional connectivity between symmetric regions of the two brain hemispheres. Preprocessed fMRI data were flipped along the midline to generate mirrored data, and the Pearson correlation coefficient was calculated between each voxel and its corresponding mirrored voxel in the opposite hemisphere. This correlation coefficient reflects the synchrony of functional activity between the two brain regions, and the data are typically further normalized using Fisher’s Z transformation to enhance normality. Intrinsic functional connectivity density (IFCD) was calculated by assessing the functional connectivity strength between each voxel and all other voxels in the brain, usually through the calculation of Pearson correlation coefficients. A threshold of 0.6 was then applied, retaining only those connections with a correlation coefficient above the threshold, and the number or density of remaining connections was counted. The IFCD value reflects the density of functional connectivity between the voxel and other regions of the brain[10,11]. All four rs-fMRI metrics (ALFF, ReHo, VMHC, and IFCD) were computed in the MNI standard space with a voxel size of 3 × 3 × 3 mm³. Each metric map was subsequently spatially smoothed using an isotropic Gaussian kernel with a full width at half maximum (FWHM) of 6 mm.

**3.3 DTI**

In this study, we focused on analyzing FW from the DTI sequence. First, the DTI data were fitted using the single-shell free water elimination diffusion tensor model, the specific equation is

$$S(g,b)=S_{0}(1-f)e^{-bg^{T}\mathrm{Dg}}+S_{0}fe^{-bD_{\mathrm{iso}}}$$

The model fitting was performed using the gradient descent method, with a total of 500 iterations and a learning rate of 0.0002. The FW results for each subject were visually inspected to confirm the convergence of the loss function curve. Subsequently, the fitted FW images were normalized to MNI152 space using the ANTs toolbox. This process included registering the individual’s DTI FA images to the FMRIB58_FA_1mm template, with a voxel size of 1x1x1 mm³[12–14]. FW represents the fractional volume of free water within each voxel, ranging from 0 to 1. Subsequent analyses were based on the mean FW values averaged across all voxels within each ROI.

**3.4 QSM**

Quantitative susceptibility mapping (QSM) preprocessing and reconstruction were performed using the QSMxT framework, which automates the entire pipeline from raw data to quantitative maps. The input data consisted of magnitude and phase images derived from a susceptibility-weighted imaging (SWI) sequence. Brain extraction was conducted using FSL’s BET with a fractional intensity threshold of 0.3. Phase unwrapping and background field removal were performed using Laplacian-based unwrapping and the V-SHARP algorithm, respectively. For dipole inversion, the Total Generalized Variation (TGV) method was applied, with regularization parameters set to their default values (e.g., α = 0.01, β = 0.2). The pipeline also incorporates the two-pass artifact reduction strategy to improve robustness in regions with phase inconsistencies. To extract susceptibility values from subcortical structures, the QSM magnitude images (1 × 1 × 1 mm³ resolution) were segmented using SynthSeg, which performs robust, deep learning-based segmentation without requiring prior preprocessing. The following bilateral regions were segmented: thalamus, caudate nucleus, putamen, globus pallidus, hippocampus, amygdala, nucleus accumbens, and ventricular cerebrospinal fluid (CSF). The mean QSM value within the ventricular CSF region was used as a reference baseline to normalize the susceptibility values of other structures, consistent with prior literature. Average QSM values for each subcortical region were extracted using the FSL toolbox, and group comparisons were subsequently conducted. To extract QSM values from ROI14, the QSM images were registered to the MNI152 standard space using ANTs (Advanced Normalization Tools) with the Symmetric Normalization (SyN) transformation model. The mean susceptibility value within ROI14 was then computed for statistical analysis[15,16].

**3.5 ASL**

In this study, CBF values were extracted from ASL sequence. First, the labeled and control images were paired, and their difference was calculated, which reflects the extent of water molecules from arterial blood entering brain tissue. Next, the M0 image was used for calibration, converting the relative signal intensity into absolute blood flow values. The CBF calculation formula accounted for several parameters, including labeling duration, T1 relaxation time of arterial blood water, labeling efficiency, and the subject’s blood and tissue characteristics. These parameters were integrated through a precise mathematical model to generate absolute CBF values for each voxel. Additionally, partial volume correction (PVC) techniques were applied to reduce the impact of gray and white matter mixing on CBF values, enhancing the accuracy of the results. The final CBF maps were then used for further quantitative analysis and group comparisons[17].

**4.** **Path analysis using structural equation modeling**

To further investigate the potential causal mechanisms linking neuroinflammation, structural alterations in cholinergic brain regions (CH123/CH4), and cognitive function, we conducted a path analysis using structural equation modeling (SEM). The model was developed based on prior theoretical assumptions and empirical evidence, incorporating free water (FW) as an index of neuroinflammation, gray matter volume (VBM) in cholinergic regions as structural markers, and multiple cognitive and functional measures, including the Mini-Mental State Examination (MMSE), Montreal Cognitive Assessment (MoCA), Auditory Verbal Learning Test (AVLT), Activities of Daily Living (ADL), and the Hamilton Depression Rating Scale (HAMD).SEM was performed using the lavaan package (version 0.6–x) in R. Standardized path coefficients were estimated using maximum likelihood estimation. Model fit was assessed using several fit indices, including the Comparative Fit Index (CFI), Tucker–Lewis Index (TLI), Root Mean Square Error of Approximation (RMSEA), and Standardized Root Mean Square Residual (SRMR). Thresholds for acceptable model fit were based on established guidelines: CFI and TLI > 0.95, RMSEA < 0.06, and SRMR < 0.08. The statistical significance of indirect (mediated) effects was evaluated using nonparametric bootstrapping with 1,000 resamples, providing bias-corrected confidence intervals and p-values for each mediation path. Due to substantial differences in variance across model variables, all continuous predictors were z-standardized prior to estimation to enhance numerical stability and minimize estimation bias.

**5.Measurement of plasma biomarkers**

Based on their critical roles in neurodegenerative pathology and neuroinflammation, we have selected Amyloid Beta 40(Aβ40), Amyloid Beta 42 (Aβ42), phosphorylated Tau protein at threonine 181(pTau181), and neurofilament light chain (NFL) as the focus of our study. Aβ42, prone to aggregation into amyloid plaques, is strongly associated with AD pathogenesis, while Aβ40 is more closely linked to vascular pathology[18]. An increased Aβ40/Aβ42 ratio may indicate early amyloid pathology in the brain and serves as a key marker for identifying the progression from MCI to AD[19]. Additionally, amyloid accumulation may disrupt neurotransmitter transmission, contributing to depressive symptoms[20]. pTau181, a sensitive marker of Tau hyperphosphorylation, reflects microtubule dysfunction and the formation of neurofibrillary tangles (NFTs), which are hallmark features of AD pathology, particularly in its early stages. Elevated plasma pTau181 levels can distinguish AD from other neurodegenerative diseases and are potentially linked to depressive symptoms through their impact on emotion-regulating regions such as the hippocampus and anterior cingulate cortex[21]. NFL, a highly sensitive indicator of axonal damage, reflects neuroinflammation or axonal degeneration. Its elevated levels have been observed in multiple neurodegenerative diseases, including AD, Parkinson’s disease, and frontotemporal dementia, and are strongly correlated with cognitive decline[22]. Additionally, NFL may reflect white matter microstructural damage, providing insights into the neuropathological mechanisms underlying depressive disorders[23]. Together, these plasma biomarkers allow for a comprehensive assessment of pathological brain changes.

Venous blood samples (2 mL) were collected from all participants under fasting conditions (7:00-9:00 AM) using EDTA-coated vacutainer tubes. Following collection, samples were centrifuged at 1,800×g for 10 min at 4°C to isolate plasma, which was then aliquoted into polypropylene cryovials (Sarstedt AG, Nümbrecht, Germany) and stored at -80°C until analysis. Hemolysis index was quantitatively assessed via spectrophotometry (414 nm absorbance >0.25 AU indicating significant hemolysis), with grossly hemolyzed specimens excluded or processed with caution.For biomarker quantification, plasma aliquots were thawed at 4°C, centrifuged at 10,000×g for 5 min, and supernatants diluted 1:4 with assay buffer before loading onto 96-well plates. Ultra-sensitive single-molecule array (Simoa) technology was implemented on the Quanterix HD-X platform (Billerica, MA, USA) using validated kits: Aβ40/Aβ42 (Neurology 3-Plex A, Cat# 101995), NfL (NF-Light Advantage Kit, Cat# 103186), and pTau181 (pTau 181 V2 Kit, Cat# 103714). Each plate included 7-point calibration curves (0.1-1000 pg/mL range) and duplicate quality controls (low/medium/high). All samples were analyzed in technical duplicates by operators blinded to clinical status, with inter-assay CVs maintained <15% across batches.

**6. The specific process of classifier training**

The modeling framework comprised two sequential phases: (1) Single-feature classification, generating 32 distinct models (4 algorithms × 8 individual features); (2) Multimodal data fusion classification, constructing 4 integrated models (4 algorithms × 1 fused dataset). All model development and evaluation procedures were implemented in Python 3.12. All neuroimaging features underwent Z-score normalization to eliminate scale disparities across modalities. Class-weighted approaches were implemented, with weights inversely proportional to class frequencies in the training set, effectively mitigating overfitting to majority classes. Leave-one-out cross-validation (LOOCV) was rigorously executed, iteratively designating single samples as test sets while utilizing remaining data for training, thereby maximizing data utility in limited cohorts and minimizing validation bias through exhaustive sampling permutations.

TABLE S1 Detailed imaging parameters for various MRI techniques

| Imaging Type | TR (ms) | TE (ms) | Flip Angle (°) | Matrix Size | FOV (mm²) |
| --- | --- | --- | --- | --- | --- |
| T1 | 2530 | 2.98 | 7 | 256×256 | 256×256 |
| DTI | 13700 | 85 | - | 112×112 | 224×224 |
| rs-fMRI | 2000 | 30 | 90 | 64×64 | 224×224 |
| ASL | 3500 | 15 | - | 64×64 | 224×224 |
| SWI | 28 | 20 | - | 320×294.4 | 240×240 |

Notes: The table outlines key scanning parameters for five different MRI modalities. Parameters such as Repetition Time (TR), Echo Time (TE), Flip Angle, Matrix Size, and Field of View (FOV) are provided for each modality.

TABLE S2 Mean values and group differences for multimodal brain region data

|  | Mean values ± SD | | | *p* value | | |
| --- | --- | --- | --- | --- | --- | --- |
|  | NC | CI-nD | CI-D | NC  vs  CI-nD | NC  vs  CI-D | CI-nD  vs  CI-D |
| VBM |  |  |  |  |  |  |
| left_ACC | 3.471±0.025 | 3.301±0.020 | 3.297±0.025 | **＜0.001** | **＜0.001** | 0.896 |
| right_ACC | 3.483±0.025 | 3.328±0.020 | 3.316±0.025 | **＜0.001** | **＜0.001** | 0.706 |
| left_AMY | 1.247±0.012 | 1.130±0.010 | 1.135±0.012 | **＜0.001** | **＜0.001** | 0.741 |
| right_AMY | 1.227±0.013 | 1.106±0.010 | 1.120±0.013 | **＜0.001** | **＜0.001** | 0.401 |
| left_CH123 | 0.059±0.0005 | 0.056±0.0004 | 0.055±0.0005 | **0.002** | **＜0.001** | 0.239 |
| right_CH123 | 0.054±0.0004 | 0.052±0.0003 | 0.051±0.0004 | **＜0.001** | **＜0.001** | 0.574 |
| left_CH4 | 0.069±0.0006 | 0.065±0.0005 | 0.065±0.0006 | **＜0.001** | **＜0.001** | 0.926 |
| right_CH4 | 0.074±0.0006 | 0.069±0.0005 | 0.070±0.0006 | **＜0.001** | **＜0.001** | 0.603 |
| left_EC | 0.784±0.008 | 0.717±0.006 | 0.720±0.008 | **＜0.001** | **＜0.001** | 0.718 |
| right_EC | 0.815±0.008 | 0.746±0.007 | 0.750±0.008 | **＜0.001** | **＜0.001** | 0.725 |
| left_HIP | 1.831±0.018 | 1.651±0.014 | 1.649±0.018 | **＜0.001** | **＜0.001** | 0.926 |
| right_HIP | 1.788±0.019 | 1.599±0.014 | 1.610±0.019 | **＜0.001** | **＜0.001** | 0.663 |
| left_PHG | 1.041±0.008 | 0.966±0.006 | 0.973±0.008 | **＜0.001** | **＜0.001** | 0.501 |
| right_PHG | 1.029±0.008 | 0.953±0.006 | 0.962±0.008 | **＜0.001** | **＜0.001** | 0.385 |
| ALFF |  |  |  |  |  |  |
| left_ACC | -0.041±0.017 | -0.019±0.017 | -0.019±0.028 | 0.066 | 0.177 | 0.889 |
| right_ACC | 0.272±0.020 | 0.322±0.020 | 0.328±0.032 | 0.29 | 0.86 | 0.609 |
| left_AMY | -0.035±0.024 | -0.001±0.024 | -0.026±0.038 | 0.28 | 0.22 | 0.62 |
| right_AMY | 0.293±0.035 | 0.356±0.035 | 0.349±0.055 | 0.87 | 0.27 | 0.24 |
| left_CH123 | 0.476±0.050 | 0.387±0.050 | 0.333±0.079 | 0.10 | 0.77 | 0.43 |
| right_CH123 | 0.497±0.050 | 0.497±0.050 | 0.406±0.080 | 0.77 | 0.45 | 0.58 |
| left_CH4 | -0.576±0.014 | -0.570±0.014 | -0.572±0.022 | 0.15 | 0.85 | 0.26 |
| right_CH4 | -0.464±0.016 | -0.455±0.016 | -0.491±0.025 | 0.45 | 0.36 | 0.80 |
| left_EC | 0.173±0.028 | 0.235±0.028 | 0.190±0.044 | 0.10 | 0.77 | 0.43 |
| right_EC | 0.721±0.042 | 0.737±0.042 | 0.785±0.067 | 0.77 | 0.45 | 0.58 |
| left_HIP | -0.500±0.010 | -0.484±0.010 | -0.508±0.017 | 0.23 | 0.71 | 0.30 |
| right_HIP | -0.400±0.011 | -0.378±0.011 | -0.404±0.018 | 0.15 | 0.85 | 0.26 |
| left_PHG | -0.246±0.014 | -0.213±0.013 | -0.220±0.022 | 0.08 | 0.36 | 0.80 |
| right_PHG | -0.107±0.017 | -0.052±0.017 | -0.074±0.027 | **0.018** | 0.34 | 0.54 |
| ReHo |  |  |  |  |  |  |
| left_ACC | -0.271±0.016 | -0.294±0.015 | -0.310±0.020 | 030 | 0.14 | 0.54 |
| right_ACC | -0.150±0.018 | -0.152±0.017 | -0.149±0.022 | 0.95 | 0.97 | 0.93 |
| left_AMY | -0.847±0.011 | -0.857±0.011 | -0.872±0.014 | 0.50 | 0.17 | 0.40 |
| right_AMY | -0.808±0.011 | -0.829±0.011 | -0.854±0.014 | 0.20 | **0.01** | 0.17 |
| left_CH123 | -0.757±0.020 | -0.746±0.019 | -0.675±0.025 | 0.68 | **0.01** | **0.028** |
| right_CH123 | -0.804±0.018 | -0.808±0.017 | -0.739±0.023 | 0.89 | **0.03** | **0.01** |
| left_CH4 | -0.798±0.014 | -0.807±0.014 | -0.817±0.018 | 0.66 | 0.42 | 0.66 |
| right_CH4 | -0.909±0.011 | -0.926±0.010 | -0.903±0.014 | 0.28 | 0.73 | 0.19 |
| left_EC | -0.893±0.016 | -0.866±0.015 | -0.910±0.020 | 0.24 | 0.53 | 0.09 |
| right_EC | -0.874±0.013 | -0.849±0.013 | -0.878±0.017 | 0.18 | 0.86 | 0.17 |
| left_HIP | -0.802±0.011 | -0.772±0.011 | -0.776±0.014 | 0.07 | 0.17 | 0.84 |
| right_HIP | -0.810±0.010 | -0.775±0.009 | -0.764±0.012 | **0.01** | **0.005** | 0.50 |
| left_PHG | -0.851±0.010 | -0.842±0.010 | -0.844±0.013 | 0.54 | 0.71 | 0.87 |
| right_PHG | -0.837±0.009 | -0.829±0.008 | -0.821±0.011 | 0.52 | 0.26 | 0.56 |
| VMHC |  |  |  |  |  |  |
| left_ACC | 0.377±0.005 | 0.381±0.005 | 0.373±0.006 | 0.574 | 0.648 | 0.333 |
| right_ACC | 0.467±0.005 | 0.470±0.005 | 0.459±0.006 | 0.663 | 0.380 | 0.197 |
| left_AMY | 0.228±0.005 | 0.223±0.005 | 0.219±0.007 | 0.490 | 0.342 | 0.715 |
| right_AMY | 0.230±0.006 | 0.226±0.005 | 0.219±0.007 | 0.631 | 0.267 | 0.474 |
| left_CH123 | 0.505±0.008 | 0.484±0.008 | 0.474±0.011 | 0.080 | 0.301 | 0.493 |
| right_CH123 | 0.606±0.122 | 0.599±0.011 | 0.584±0.015 | 0.687 | 0.277 | 0.446 |
| left_CH4 | 0.154±0.007 | 0.142±0.007 | 0.163±0.009 | 0.261 | 0.471 | 0.082 |
| right_CH4 | 0.147±0.006 | 0.137±0.008 | 0.159±0.008 | 0.301 | 0.233 | **0.032** |
| left_EC | 0.180±0.005 | 0.182±0.005 | 0.168±0.006 | 0.739 | 0.188 | 0.099 |
| right_EC | 0.162±0.005 | 0.166±0.004 | 0.155±0.006 | 0.582 | 0.421 | 0.188 |
| left_HIP | 0.204±0.004 | 0.199±0.004 | 0.194±0.005 | 0.372 | 0.161 | 0.515 |
| right_HIP | 0.198±0.004 | 0.196±0.004 | 0.192±0.005 | 0.804 | 0.393 | 0.510 |
| left_PHG | 0.235±0.005 | 0.234±0.004 | 0.229±0.006 | 0.910 | 0.483 | 0.543 |
| right_PHG | 0.172±0.004 | 0.167±0.004 | 0.166±0.005 | 0.477 | 0.421 | 0.483 |
| IFCD |  |  |  |  |  |  |
| left_ACC | -0.208±0.011 | -0.197±0.011 | -0.197±0.014 | 0.511 | 0559 | 0.986 |
| right_ACC | -0.126±0.015 | -0.109±0.014 | -0.118±0.018 | 0.407 | 0.747 | 0.687 |
| left_AMY | -0.493±0.007 | -0.464±0.007 | -0.467±0.009 | 0.009 | **0.049** | 0.7774 |
| right_AMY | -0.440±0.006 | -0.426±0.008 | -0.428±0.008 | 0.144 | 0.264 | 0.883 |
| left_CH123 | -0.406±0.011 | -0.425±0.010 | -0.432±0.013 | 0.208 | 0.149 | 0.713 |
| right_CH123 | -0.386±0.007 | -0.396±0.007 | -0.397±0.009 | 0.334 | 0.385 | 0.962 |
| left_CH4 | -0.505±0.006 | -0.496±0.006 | -0.496±0.008 | 0.366 | 0.419 | 0.713 |
| right_CH4 | -0.511±0.006 | -0.508±0.006 | -0.496±0.008 | 0.301 | 0.233 | **0.032** |
| left_EC | -0.061±0.001 | -0.059±0.008 | -0.059±0.001 | 0.956 | 0.627 | 0.823 |
| right_EC | -0.017±0.0002 | -0.016±0.0002 | -0.016±0.0003 | 0.776 | 0.673 | 0.492 |
| left_HIP | -0.513±0.007 | -0.496±0.007 | -0.502±0.009 | 0.117 | 0.310 | 0.730 |
| right_HIP | 0.512±0.006 | 0.503±0.006 | 0.499±0.008 | 0.173 | 0.440 | 0.680 |
| left_PHG | 0.411±0.005 | 0.394±0.005 | 0.402±0.007 | 0.100 | 0.363 | 0.600 |
| right_PHG | 0.388±0.005 | 0.375±0.005 | 0.382±0.006 | 0.337 | 0.254 | 0.746 |
| CBF |  |  |  |  |  |  |
| left_ACC | 44.91±1.108 | 45.62±0.970 | 46.37±1.242 | 0.614 | 0.367 | 0.635 |
| right_ACC | 46.69±1.086 | 47.62±1.036 | 49.09±1.326 | 0.533 | 0.167 | 0.384 |
| left_AMY | 41.24±1.156 | 42.12±1.102 | 45.01±1.410 | 0.582 | **0.04** | 0.106 |
| right_AMY | 42.25±1.088 | 41.44±1.037 | 44.71±1.327 | 0.592 | 0.155 | 0.053 |
| left_CH123 | 44.23±1.154 | 45.59±1.101 | 48.19±1.408 | 0.396 | **0.031** | 0.146 |
| right_CH123 | 42.83±1.206 | 44.05±1.150 | 46.40±1.471 | 0.466 | 0.062 | 0.207 |
| left_CH4 | 53.91±1.462 | 57.94±1.395 | 60.31±1.785 | **0.047** | **0.005** | 0.282 |
| right_CH4 | 56.76±1.482 | 57.42±1.414 | 61.40±1.809 | 0.748 | **0.049** | 0.083 |
| left_EC | 57.76±1.997 | 56.00±1.905 | 58.10±2.437 | 0.522 | 0.917 | 0.497 |
| right_EC | 62.26±2.093 | 57.31±1.996 | 57.48±2.554 | 0.748 | **0.049** | 0.083 |
| left_HIP | 62.60±1.163 | 63.10±1.109 | 65.094±1.419 | 0.756 | 0.177 | 0.269 |
| right_HIP | 66.04±1.223 | 63.62±1.166 | 68.14±1.492 | 0.153 | 0.279 | 0.017 |
| left_PHG | 67.24±1.328 | 68.99±1.266 | 70.90±1.620 | 0.341 | 0.082 | 0.353 |
| right_PHG | 71.55±1.407 | 68.78±1.342 | 75.23±1.717 | 0.153 | 0.100 | **0.003** |
| QSM |  |  |  |  |  |  |
| left_ACC | 0.0004±0.0001 | 0.0005±0.0001 | 0.0008±0.0001 | 0.503 | **0.034** | 0.112 |
| right_ACC | 0.0001±0.0001 | 0.0004±0.0001 | 0.0007±0.0001 | 0.079 | **0.004** | 0.079 |
| left_AMY | 0.0009±0.0001 | 0.0013±0.0001 | 0.0012±0.0002 | 0.110 | 0.354 | 0.628 |
| right_AMY | 0.0003±0.0001 | 0.0009±0.0001 | 0.0008±0.0002 | **0.022** | 0.090 | 0.751 |
| left_CH123 | 0.005±0.0006 | 0.007±0.0006 | 0.008±0.0008 | **0.046** | **0.017** | 0.512 |
| right_CH123 | 0.005±0.0006 | 0.005±0.0006 | 0.006±0.0007 | 0.507 | 0.441 | 0.843 |
| left_CH4 | 0.003±0.0005 | 0.004±0.0005 | 0.004±0.0006 | 0.752 | 0.717 | 0.509 |
| right_CH4 | 0.004±0.0005 | 0.004±0.0004 | 0.003±0.0006 | 0.998 | 0.122 | 0.109 |
| left_EC | -0.0029±0.0002 | -0.0029±0.0002 | -0.0021±0.0002 | 0.998 | **0.033** | **0.028** |
| right_EC | -0.0025±0.0001 | -0.0030±0.0001 | -0.0025±0.0002 | **0.038** | 0.941 | 0.072 |
| left_HIP | 0.0004±0.0001 | 0.0004±0.0001 | 0.0007±0.0001 | 0.890 | 0.073 | 0.748 |
| right_HIP | 0.0004±0.0001 | 0.0007±0.0001 | 0.0009±0.0001 | 0.091 | **0.008** | 0.216 |
| left_PHG | -0.0002±0.0001 | 0.00003±0.0001 | 0.0002±0.0001 | 0.183 | **0.040** | 0.354 |
| right_PHG | 0.0006±0.0001 | 0.001±0.0001 | 0.001±0.0002 | **0.028** | **0.015** | 0.600 |
| FW |  |  |  |  |  |  |
| left_ACC | 0.352±0.002 | 0.368±0.002 | 0.378±0.003 | **＜0.001** | **＜0.001** | **0.04** |
| right_ACC | 0.419±0.002 | 0.441±0.003 | 0.446±0.004 | **＜0.001** | **＜0.001** | 0.36 |
| left_AMY | 0.374±0.004 | 0.402±0.004 | 0.410±0.005 | **＜0.001** | **＜0.001** | 0.24 |
| right_AMY | 0.420±0.005 | 0.451±0.005 | 0.457±0.006 | **＜0.001** | **＜0.001** | 0.51 |
| left_CH123 | 0.529±0.006 | 0.534±0.006 | 0.561±0.008 | 0.59 | **0.004** | **0.013** |
| right_CH123 | 0.666±0.008 | 0.687±0.008 | 0.709±0.010 | 0.07 | **0.01** | 0.09 |
| left_CH4 | 0.582±0.005 | 0.580±0.005 | 0.605±0.007 | 0.82 | **0.014** | **0.006** |
| right_CH4 | 0.628±0.006 | 0.637±0.006 | 0.662±0.008 | 0.35 | **0.001** | **0.014** |
| left_EC | 0.410±0.005 | 0.440±0.005 | 0.450±0.006 | **＜0.001** | **＜0.001** | 0.24 |
| right_EC | 0.385±0.007 | 0.407±0.007 | 0.422±0.009 | **0.04** | **0.003** | 0.22 |
| left_HIP | 0.356±0.007 | 0.496±0.007 | 0.514±0.009 | **＜0.001** | **＜0.001** | 0.13 |
| right_HIP | 0.339±0.008 | 0.512±0.007 | 0.525±0.009 | **＜0.001** | **＜0.001** | 0.33 |
| left_PHG | 0.280±0.007 | 0.422±0.006 | 0.428±0.008 | **＜0.001** | **＜0.001** | 0.60 |
| right_PHG | 0.289±0.009 | 0.501±0.009 | 0.507±0.011 | **＜0.001** | **＜0.001** | 0.70 |

Notes: The table presents mean values (± SD) for various brain regions across different groups: NC, CI-nD, and CI-D, *p*-values indicate the statistical significance of differences between groups for each brain region, *p＜0*.05. Brain regions include the ACC, AMY, BF4, BF123, EC, HIP, and PHG.

TABLE S3 Statistically significant associations between multimodal imaging features and blood biomarkers in the CI-nD group

| Blood biomarkers | Imaging features | Correlation coefficient | *p* |
| --- | --- | --- | --- |
| **NFL** | **FW-right-HIP** | **0.405** | **0.003** |
| **ptau181** | **FW-left-HIP** | **0.376** | **0.006** |
| **Aβ40** | **ReHo- right-AMY** | **0.339** | **0.014** |
| NFL | QSM-right-PHG | -0.335 | 0.015 |
| Aβ40 | lFCD- right-AMY | 0.334 | 0.015 |
| Aβ40 | FW- right-PHG | -0.317 | 0.022 |
| Aβ42 | QSM-left-EC | 0.314 | 0.023 |
| Aβ42 | VMHC-left-CH123 | -0.309 | 0.026 |
| Aβ40 | QSM- right-EC | -0.308 | 0.026 |
| ptau181 | FW- right-CH4 | 0.308 | 0.026 |
| ptau181 | QSM- right-AMY | 0.305 | 0.028 |
| ptau181 | FW- left-CH123 | 0.301 | 0.030 |
| Aβ40 | lFCD- right-EC | 0.289 | 0.038 |
| NFL | VMHC-right-CH123 | -0.279 | 0.045 |
| NFL | VMHC- left-HIP | -0.277 | 0.047 |

TABLE S4 Statistically significant associations between multimodal imaging features and blood biomarkers in the CI-D group

| Blood biomarkers | Imaging features | Correlation coefficient | *p* |
| --- | --- | --- | --- |
| **NFL** | **VMHC-right-CH123** | **-0.704** | **0.002** |
| **NFL** | **lFCD- right-EC** | **-0.684** | **0.003** |
| **Aβ40** | **FW-left-CH4** | **-0.669** | **0.005** |
| **Aβ42** | **VMHC- right-ACC** | **0.669** | **0.005** |
| **NFL** | **lFCD- right-CH4** | **-0.669** | **0.005** |
| NFL | lFCD-right-HIP | -0.656 | 0.006 |
| ptau181 | ALFF- right-CH123 | 0.622 | 0.010 |
| ptau181 | ReHo- right-EC | 0.618 | 0.011 |
| ptau181 | lFCD- right-ACC | 0.610 | 0.012 |
| ptau181 | ReHo-left-AMY | 0.600 | 0.014 |
| Aβ42 | VMHC- left-ACC | 0.584 | 0.018 |
| Aβ40 | lFCD- left-CH123 | -0.581 | 0.018 |
| NFL | lFCD- right-AMY | -0.577 | 0.019 |
| NFL | lFCD- right-PHG | -0.573 | 0.020 |
| Aβ40 | CBF- right-HIP | 0.561 | 0.024 |
| Aβ40 | lFCD- left-HIP | -0.559 | 0.024 |
| NFL | lFCD- left-CH4 | -0.556 | 0.025 |
| NFL | CBF- left-HIP | -0.553 | 0.026 |
| ptau181 | VMHC- left-EC | 0.531 | 0.034 |
| NFL | lFCD- left-HIP | -0.530 | 0.035 |
| NFL | lFCD- right-CH123 | -0.507 | 0.045 |
| NFL | lFCD- left-PHG | -0.506 | 0.046 |
| AB40 | FW- left-CH123 | -0.503 | 0.047 |
| NFL | CBF- left-CH4 | -0.503 | 0.047 |
| ptau181 | ReHo- right-AMY | 0.501 | 0.048 |
| ptau181 | ALFF- left-CH123 | 0.498 | 0.049 |

Notes: The table presents Spearman’s correlation analysis between blood biomarkers and imaging features revealed that in the CI-nD and CI-D. *p* < 0.05 indicates a significant correlation between imaging features and plasma biomarkers. The bolded sections represent the imaging features that are in the top 20% of correlations with plasma biomarkers.

Table S5 Comparative performance of SVM models under multimodal fusion and single-modality neuroimaging metrics

| Feature | Accuracy | Sensitivity | Specificity | AUC |
| --- | --- | --- | --- | --- |
| ALFF | 0.60 | 1.00/0.73/0.10 | 1.00/0.63/0.88 | 1.00/0.71/0.72 |
| ReHo | 0.74 | 1.00/0.78/0.16 | 1.00/0.77/0.22 | 1.00/0.77/0.74 |
| IFCD | 0.66 | 1.00/0.82/0.14 | 1.00/0.77/0.60 | 1.00/0.88/0.78 |
| VMHC | 0.70 | 1.00/0.73/0.17 | 1.00/0.77/0.32 | 1.00/0.76/0.75 |
| GMV | 0.66 | 1.00/0.82/0.14 | 1.00/0.77/0.59 | 0.89/0.72/0.73 |
| FW | 0.67 | 1.00/0.88/0.16 | 1.00/0.67/0.67 | 1.00/0.80/0.79 |
| QSM | 0.66 | 1.00/0.66/0.33 | 1.00/0.73/0.85 | 1.00/0.80/0.79 |
| CBF | 0.71 | 1.00/0.75/0.28 | 1.00/0.73/0.59 | 1.00/0.78/0.74 |
| Multimodal fusion | 0.85 | 0.90/0.82/0.81 | 0.88/0.93/0.95 | 0.95/0.96/0.96 |

**Note:** Sensitivity, specificity, and AUC values are listed in the order of NC, CI-nD, and CI-D groups, separated by slashes ("/").

Table S6 Comparative performance of LR models under multimodal fusion and single-modality neuroimaging metrics

| Feature | Accuracy | Sensitivity | Specificity | AUC |
| --- | --- | --- | --- | --- |
| ALFF | 0.67 | 1.00/0.75/0.10 | 1.00/0.63/0.89 | 1.00/0.71/0.72 |
| ReHo | 0.72 | 1.00/0.88/0.13 | 1.00/0.65/0.14 | 1.00/0.77/0.76 |
| IFCD | 0.70 | 1.00/0.78/0.17 | 1.00/0.77/0.11 | 1.00/0.76/0.76 |
| VMHC | 0.71 | 1.00/0.84/0.13 | 1.00/0.64/0.93 | 1.00/0.54/0.52 |
| GMV | 0.72 | 1.00/0.88/0.22 | 1.00/0.69/0.60 | 1.00/0.79/0.77 |
| FW | 0.71 | 1.00/0.81/0.17 | 1.00/0.77/0.66 | 1.00/0.78/0.78 |
| QSM | 0.72 | 1.00/0.84/0.17 | 1.00/0.77/0.77 | 1.00/0.79/0.78 |
| CBF | 0.65 | 0.84/0.81/0.20 | 0.12/0.20/0.65 | 0.94/0.77/0.76 |
| Multimodal fusion | 0.68 | 0.84/0.97/0.7 | 0.12/0.45/0.77 | 0.94/0.83/0.78 |

Table S7 Comparative performance of CatBoost models under multimodal fusion and single-modality neuroimaging metrics

| Feature | Accuracy | Sensitivity | Specificity | AUC |
| --- | --- | --- | --- | --- |
| ALFF | 0.70 | 1.00/0.75/0.20 | 1.00/0.67/0.89 | 1.00/0.79/0.79 |
| ReHo | 0.69 | 1.00/0.69/0.23 | 1.00/0.68/0.34 | 1.00/0.77/0.76 |
| IFCD | 0.70 | 1.00/0.78/0.17 | 1.00/0.77/0.31 | 1.00/0.76/0.75 |
| VMHC | 0.75 | 1.00/0.66/0.47 | 1.00/0.78/0.56 | 1.00/0.83/0.82 |
| GMV | 0.73 | 1.00/0.75/0.73 | 1.00/0.79/0.50 | 1.00/0.83/0.82 |
| FW | 0.71 | 1.00/0.66/0.33 | 1.00/0.73/0.85 | 1.00/0.80/0.79 |
| QSM | 0.73 | 1.00/0.75/0.30 | 1.00/0.73/0.66 | 1.00/0.80/0.79 |
| CBF | 0.72 | 1.00/0.75/0.27 | 1.00/0.73/0.64 | 1.00/0.77/0.76 |
| Multimodal fusion | 0.75 | 1.00/0.88/0.23 | 1.00/0.75/0.59 | 1.00/0.77/0.76 |

Table S8 Comparative performance of RF models under multimodal fusion and single-modality neuroimaging metrics

| Feature | Accuracy | Sensitivity | Specificity | AUC |
| --- | --- | --- | --- | --- |
| ALFF | 0.69 | 1.00/0.75/0.17 | 1.00/0.66/0.89 | 1.00/0.79/0.78 |
| ReHo | 0.73 | 1.00/0.88/0.17 | 1.00/0.67/0.78 | 1.00/0.80/0.79 |
| IFCD | 0.72 | 1.00/0.88/0.13 | 1.00/0.69/0.67 | 1.00/0.79/0.77 |
| VMHC | 0.75 | 1.00/0.75/0.37 | 1.00/0.59/0.45 | 1.00/0.86/0.84 |
| GMV | 0.75 | 1.00/0.88/0.23 | 1.00/0.67/0.79 | 1.00/0.83/0.81 |
| FW | 0.75 | 1.00/0.88/0.27 | 1.00/0.78/0.69 | 1.00/0.83/0.82 |
| QSM | 0.74 | 1.00/0.81/0.27 | 1.00/0.68/0.67 | 1.00/0.80/0.76 |
| CBF | 0.68 | 1.00/0.81/0.26 | 1.00/0.58/0.77 | 1.00/0.80/0.79 |
| Multimodal fusion | 0.77 | 1.00/0.91/0.30 | 1.00/0.60/0.67 | 1.00/0.78/0.80 |

FIGURE S1 Preprocessing pipeline for multimodal neuroimaging data.


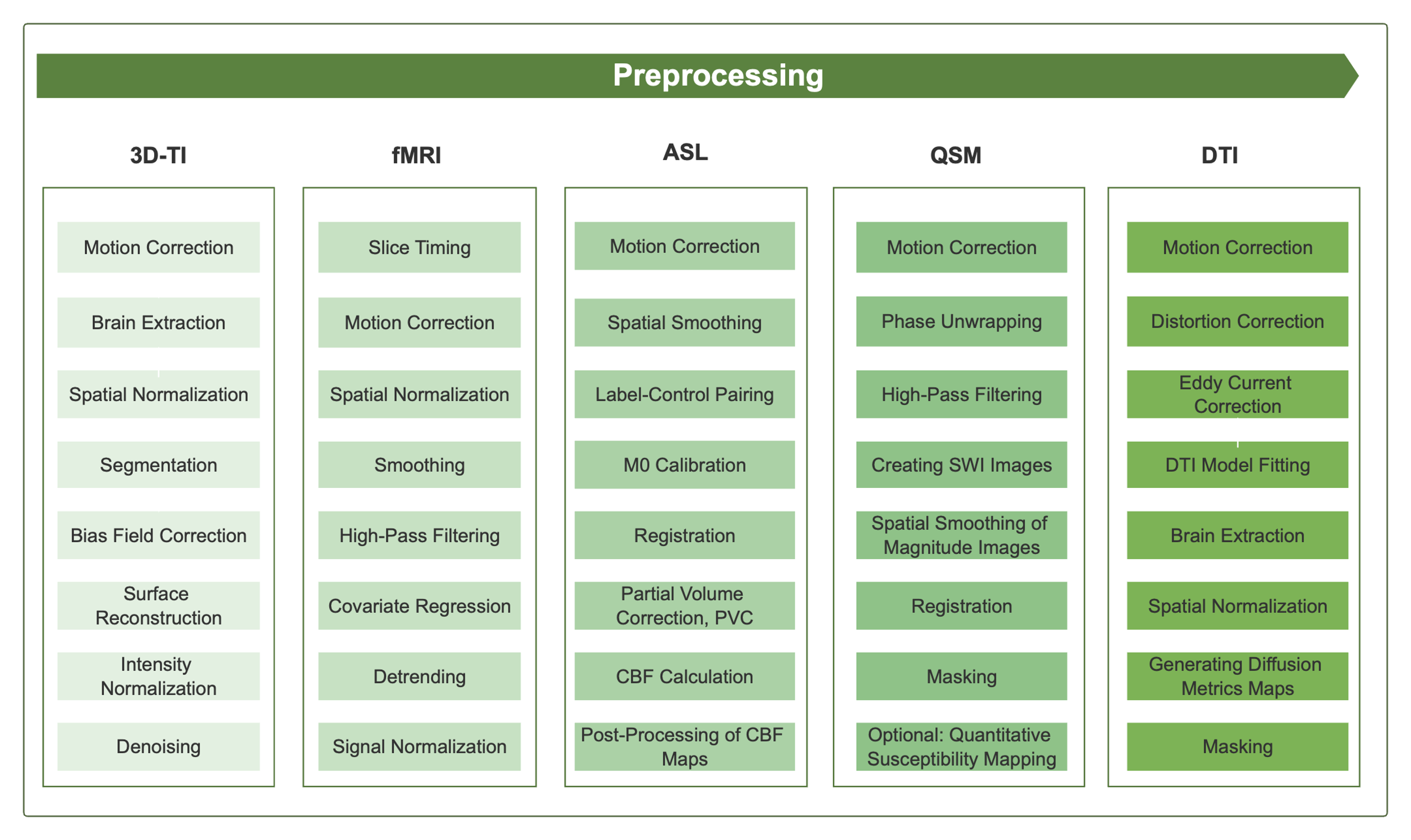
Note: This diagram presents the preprocessing steps applied to different neuroimaging modalities: 3D-TI, fMRI, ASL, QSM, and DTI. Each modality has its own tailored set of preprocessing operations to ensure that the data is normalized, corrected, and prepared for further analysis.

FIGURE S2


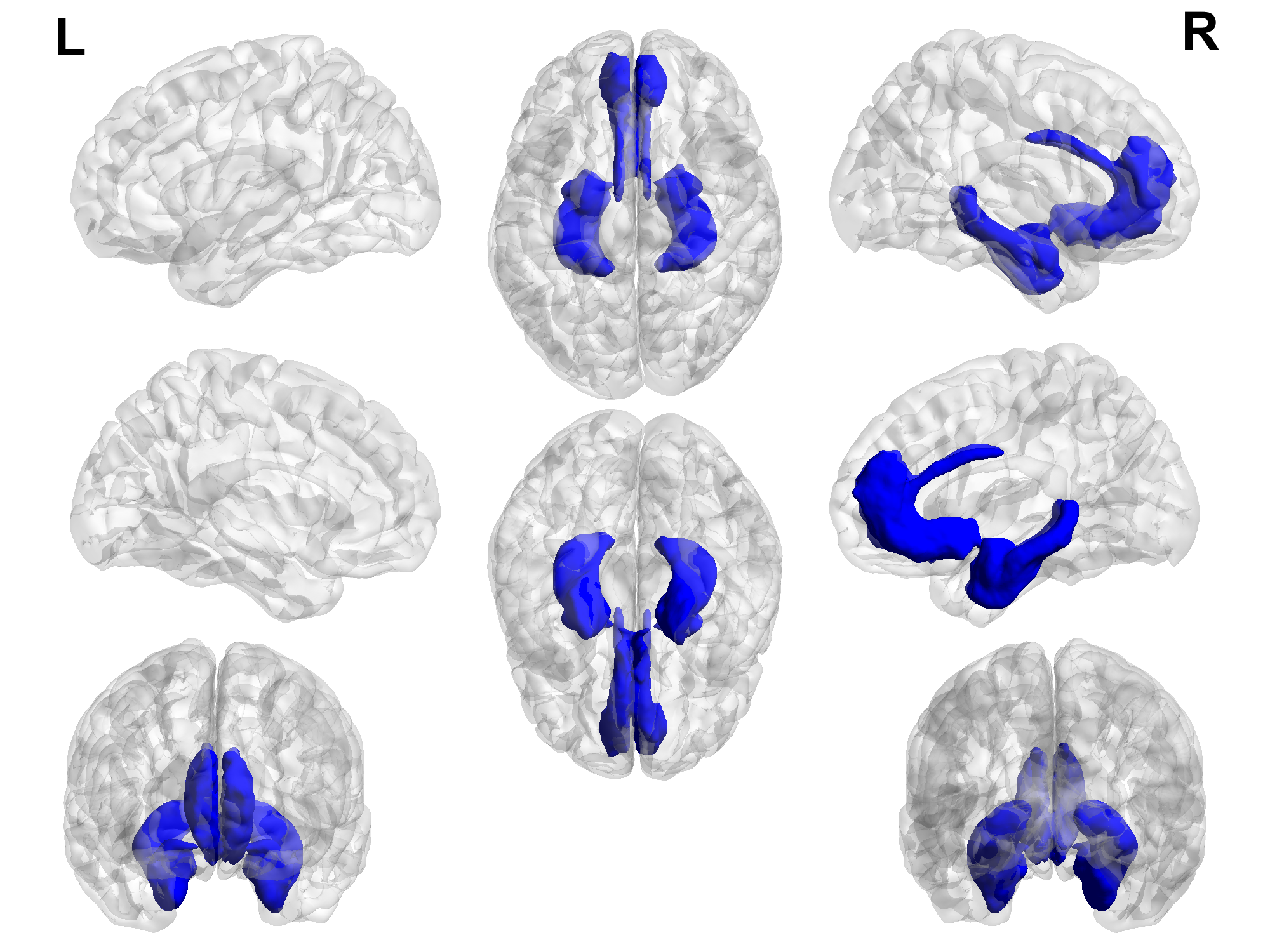


(1) Gray matter volume reductions in CI-nD and CI-D groups compared to NC


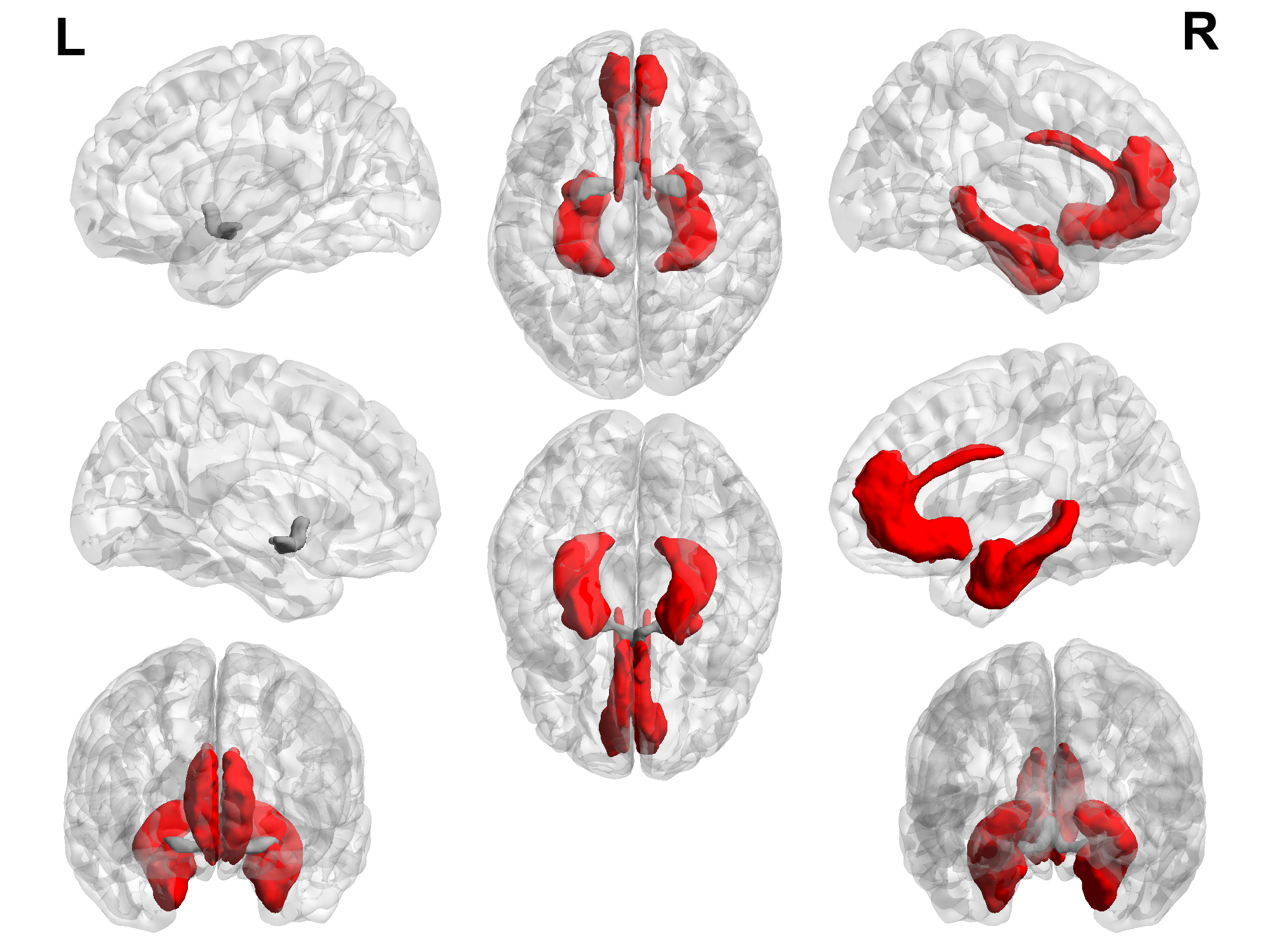


(2) FW elevation in CI-nD group compared to NC group


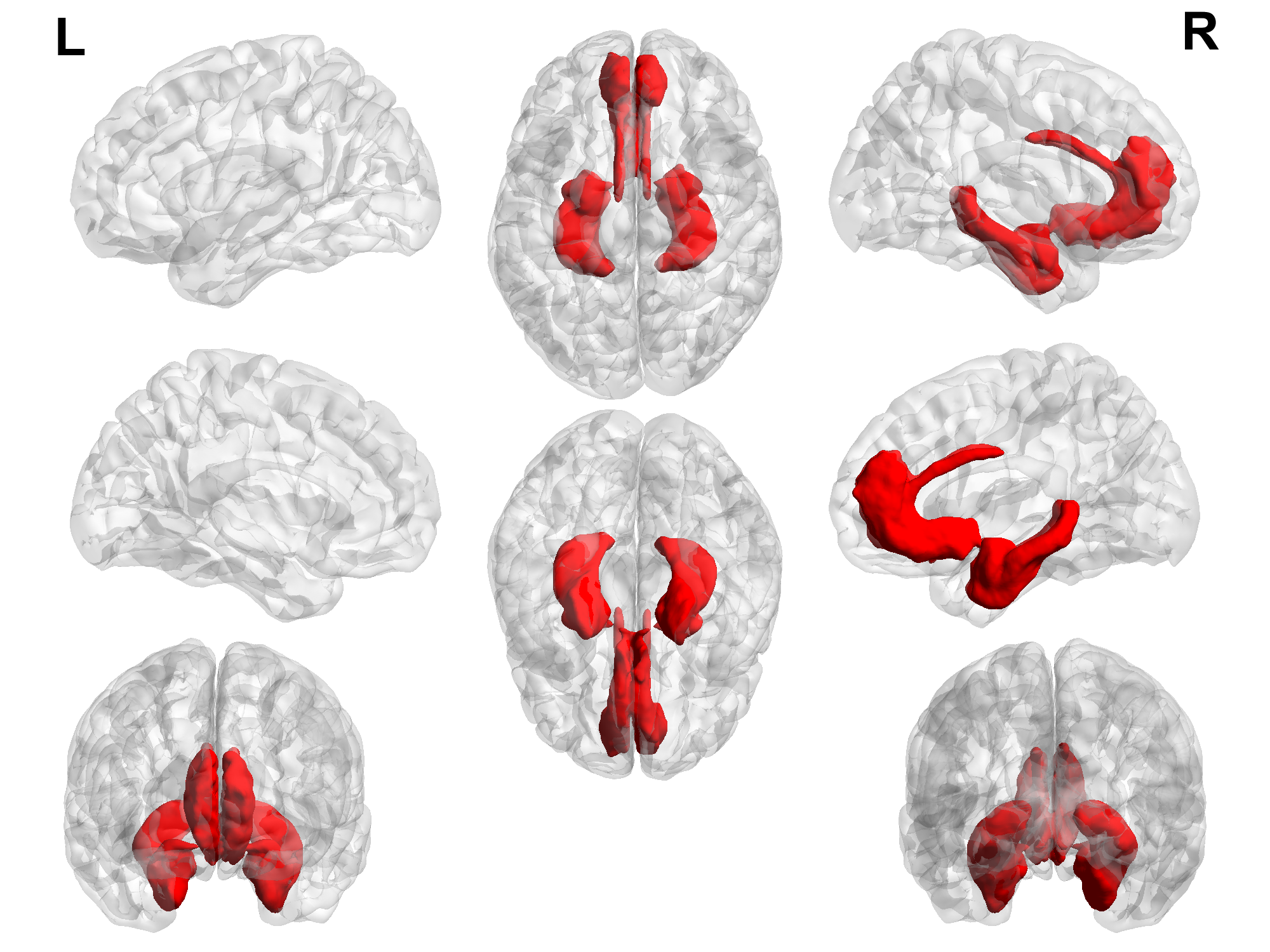


(3) FW elevation in CI-D group compared to NC group


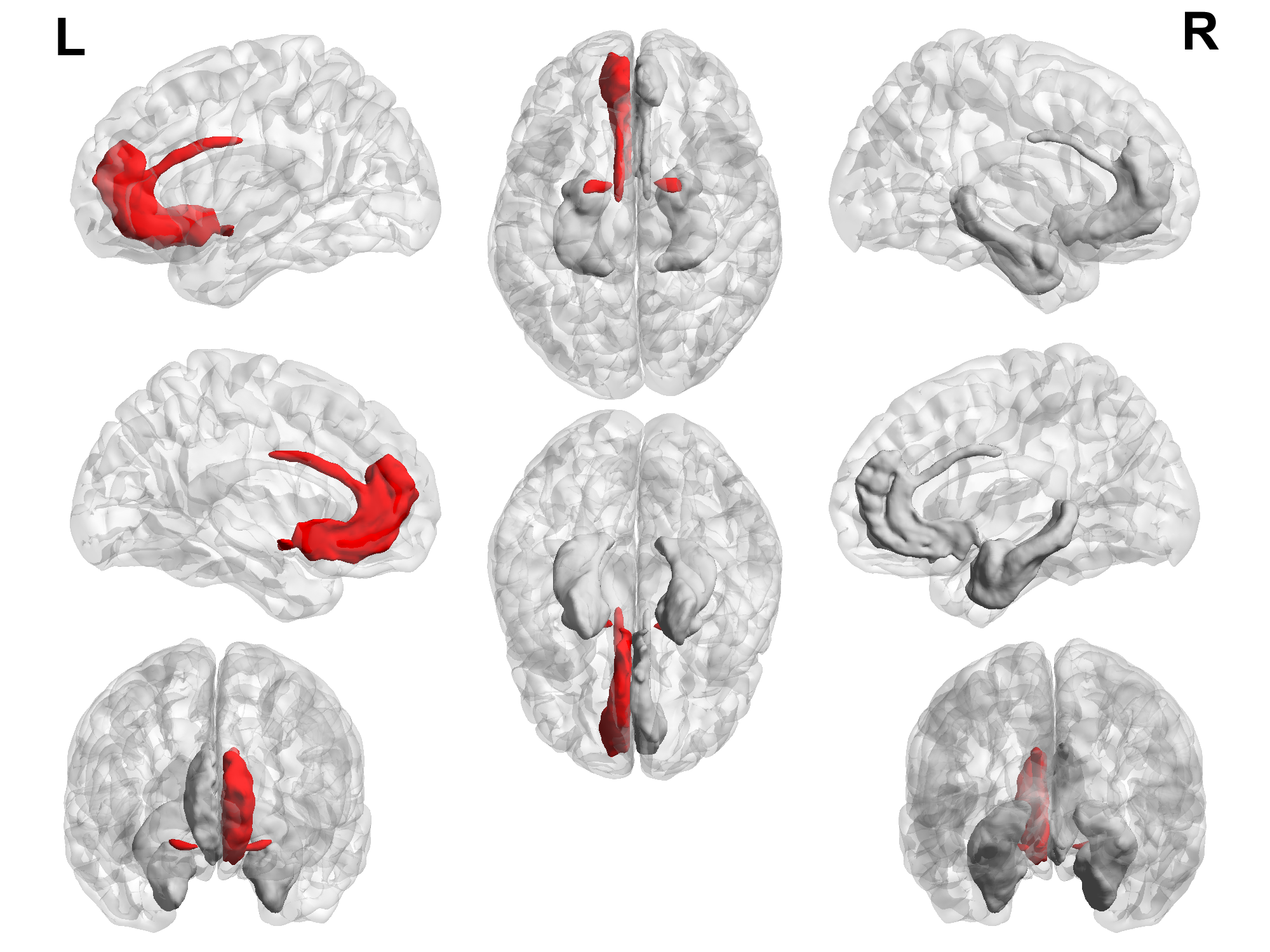


(4) FW elevation in CI-D group compared to CI-nD group


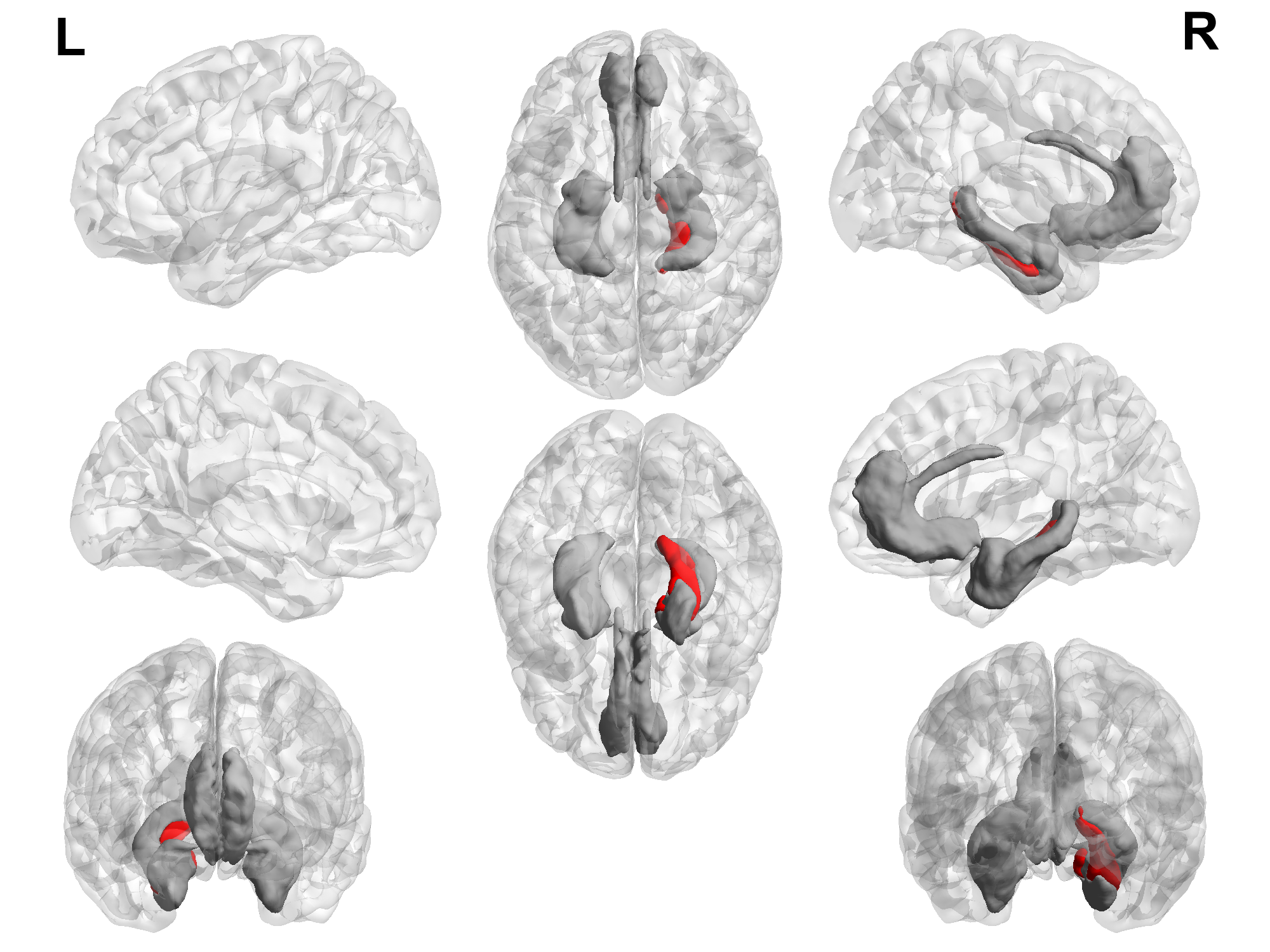


(5) ALFF elevation in CI-nD group compared to NC group


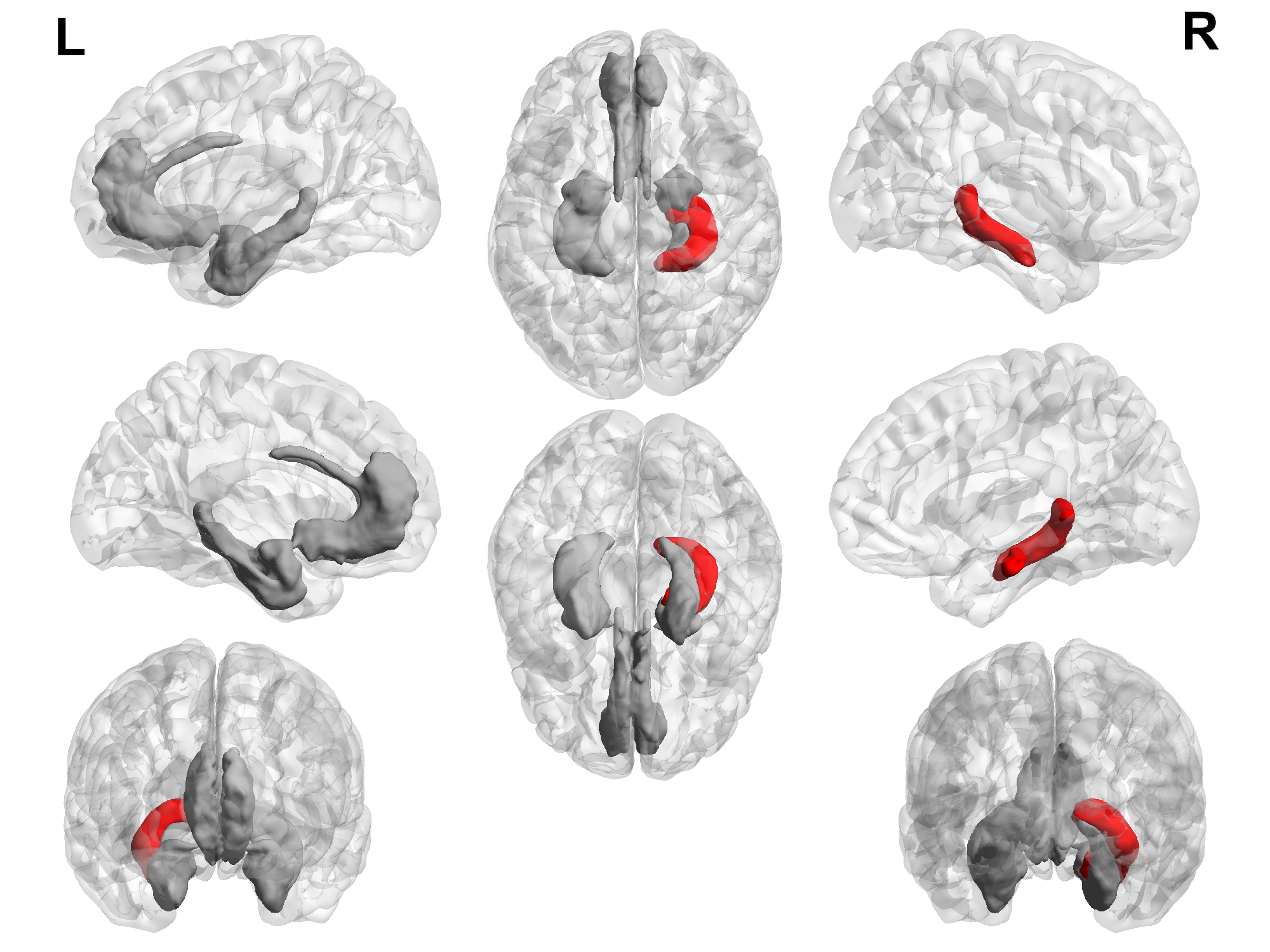


(6) ReHo elevation in CI-nD group compared to NC group


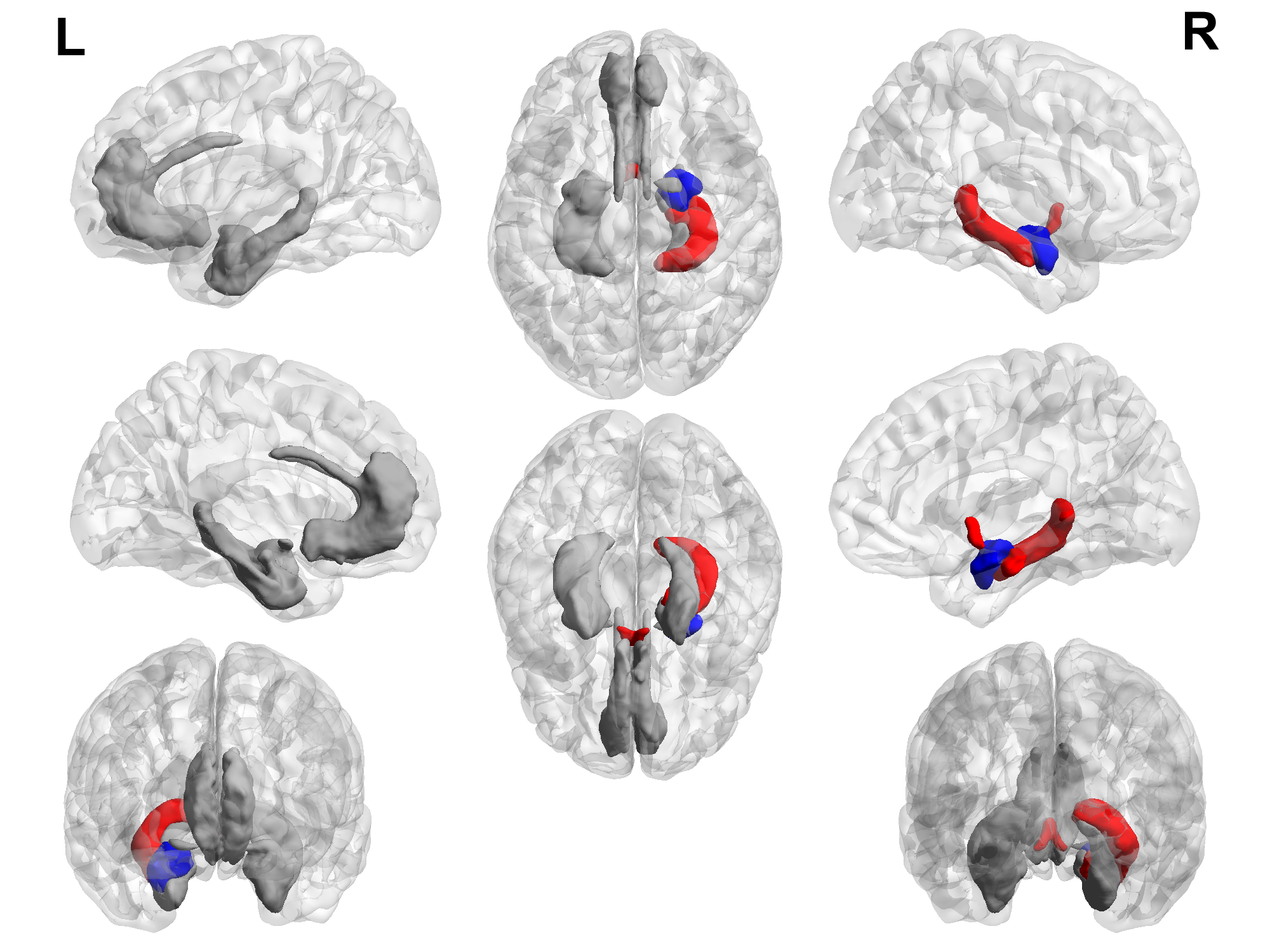


(7) ReHo elevation and reduction in CI-D group compared to NC group


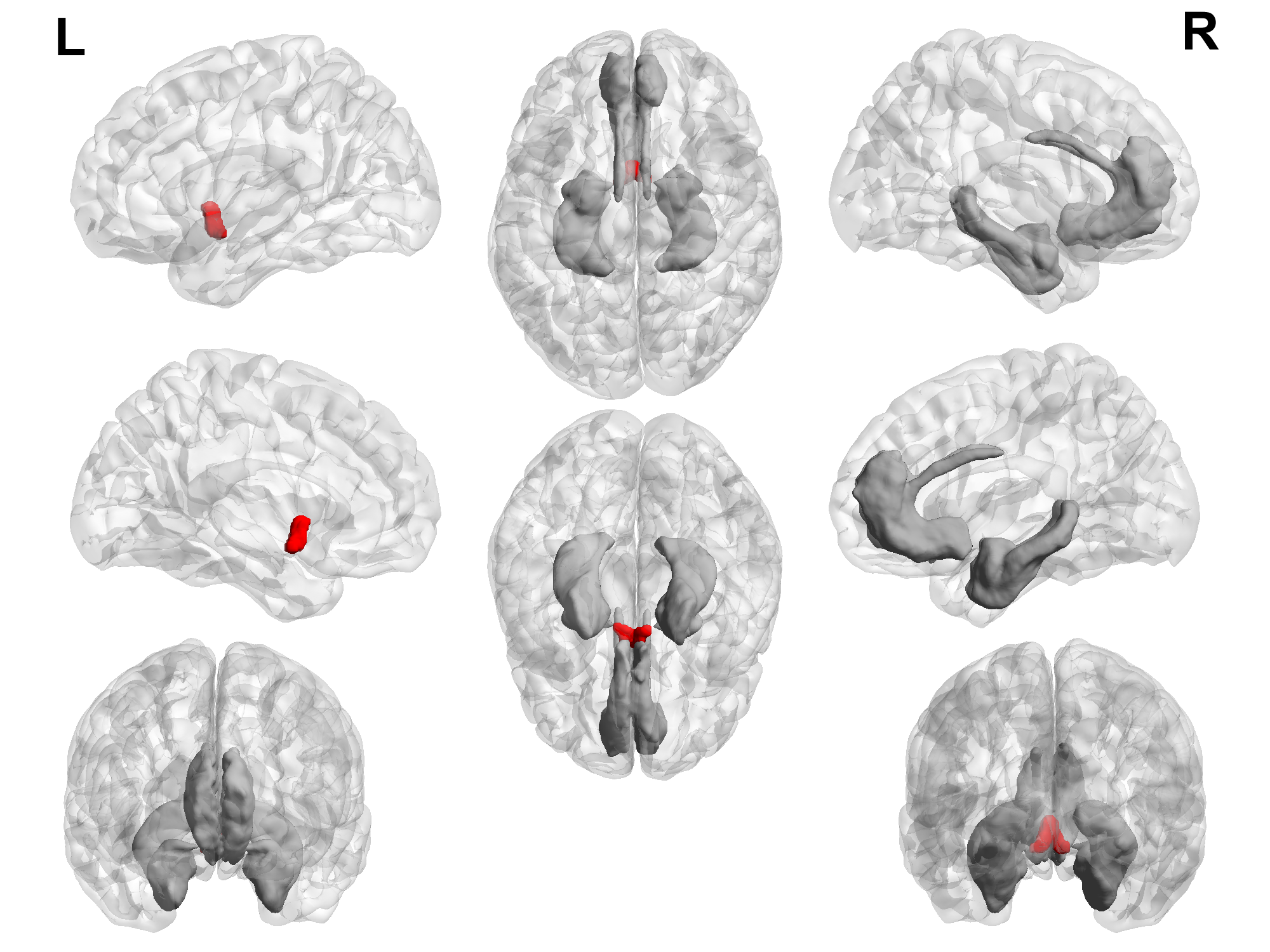


(8) ReHo elevation in CI-D group compared to CI-nD group


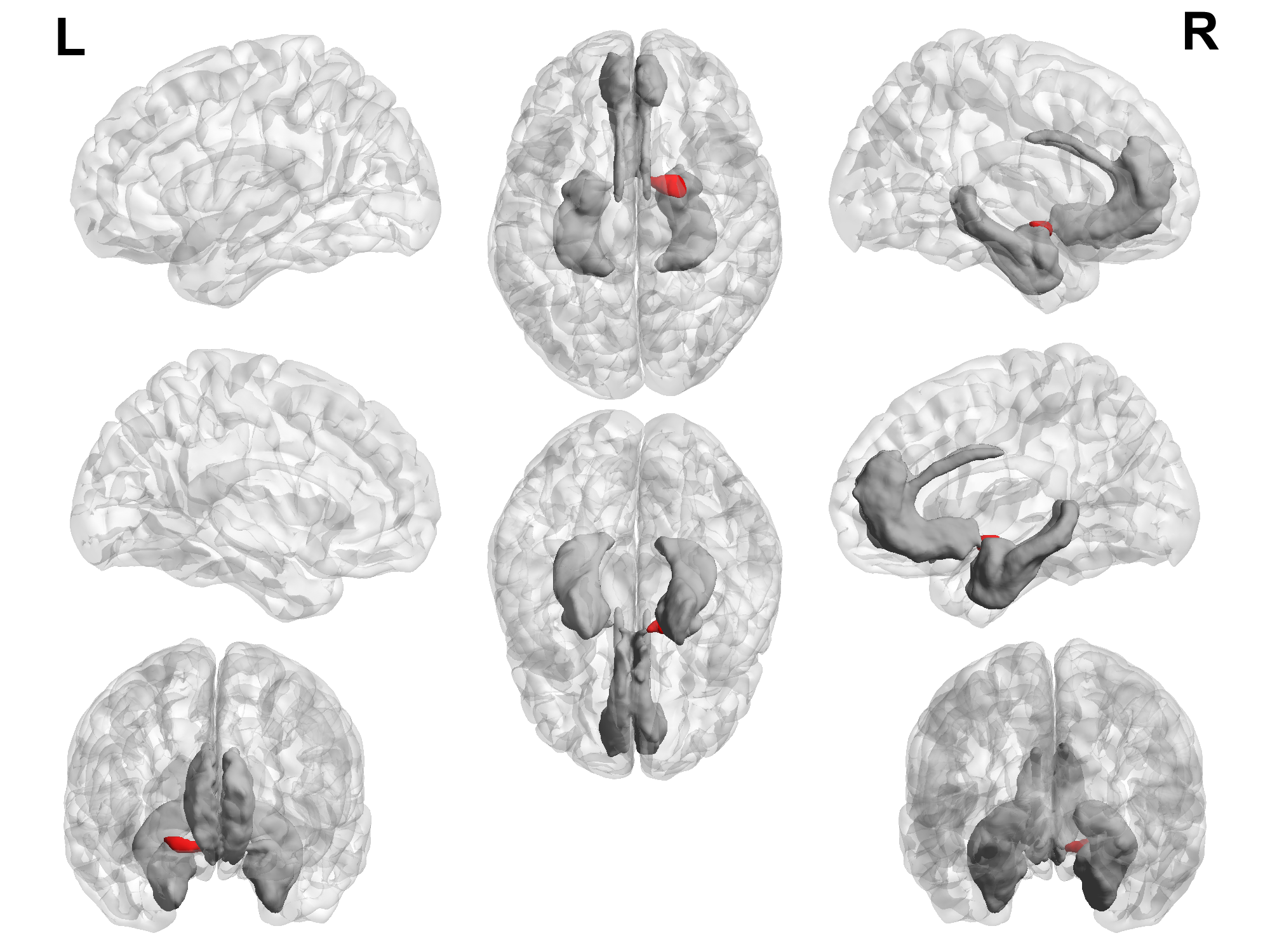


(9) VMHC elevation in CI-D group compared to CI-nD group


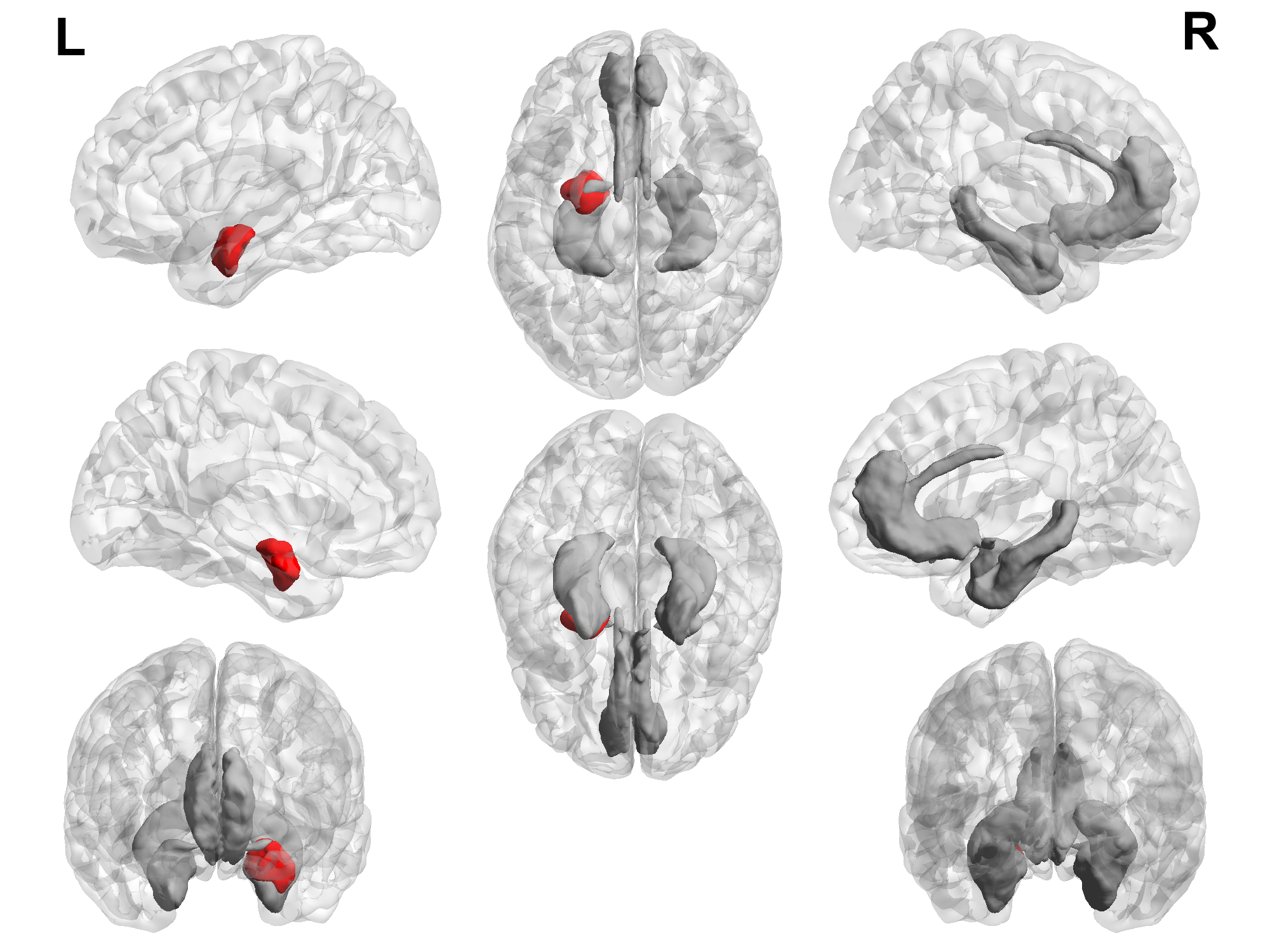


(10) IFCD elevation in CI-D group compared to NC group


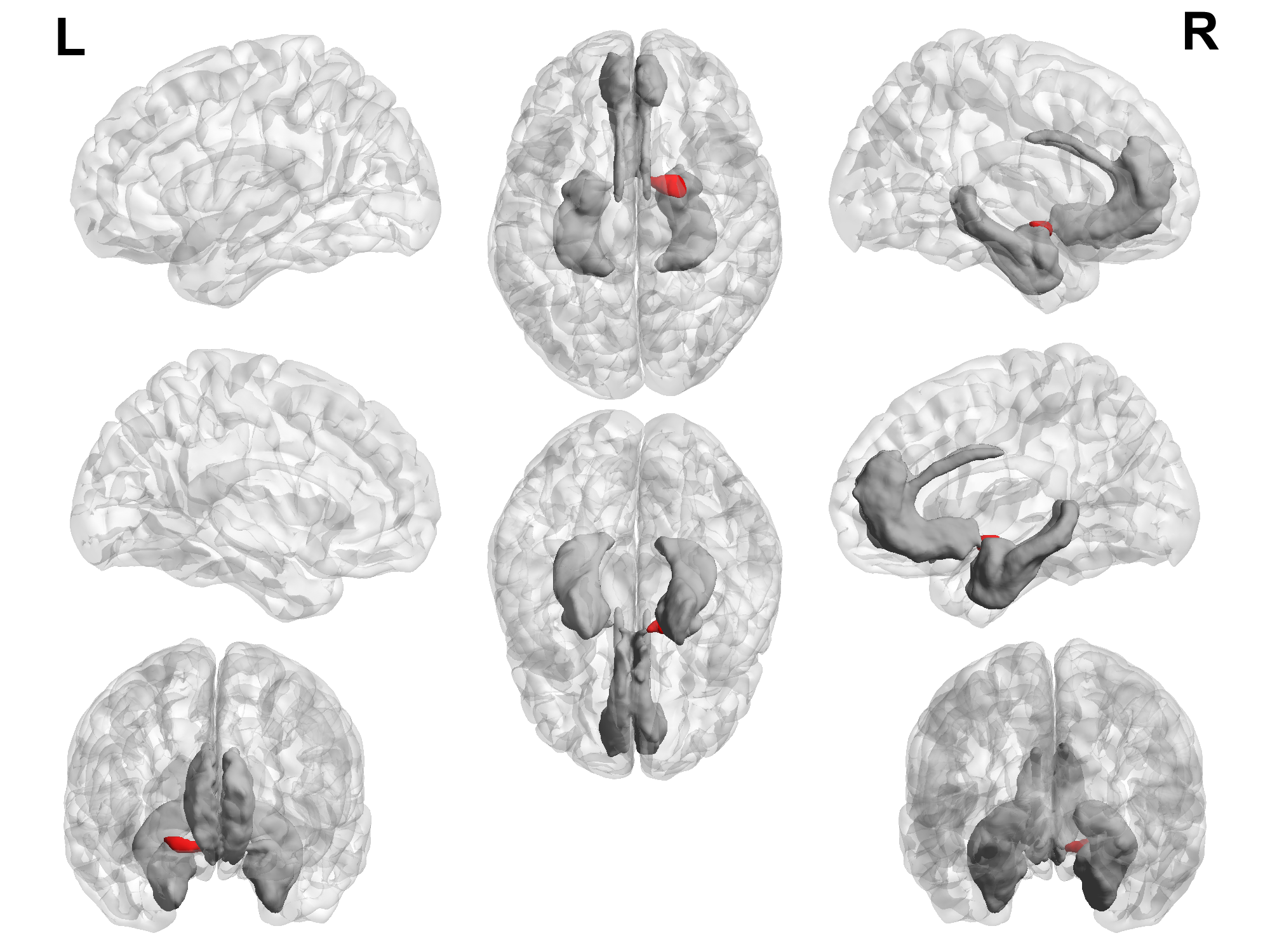


(11) IFCD elevation in CI-D group compared to CI-nD group


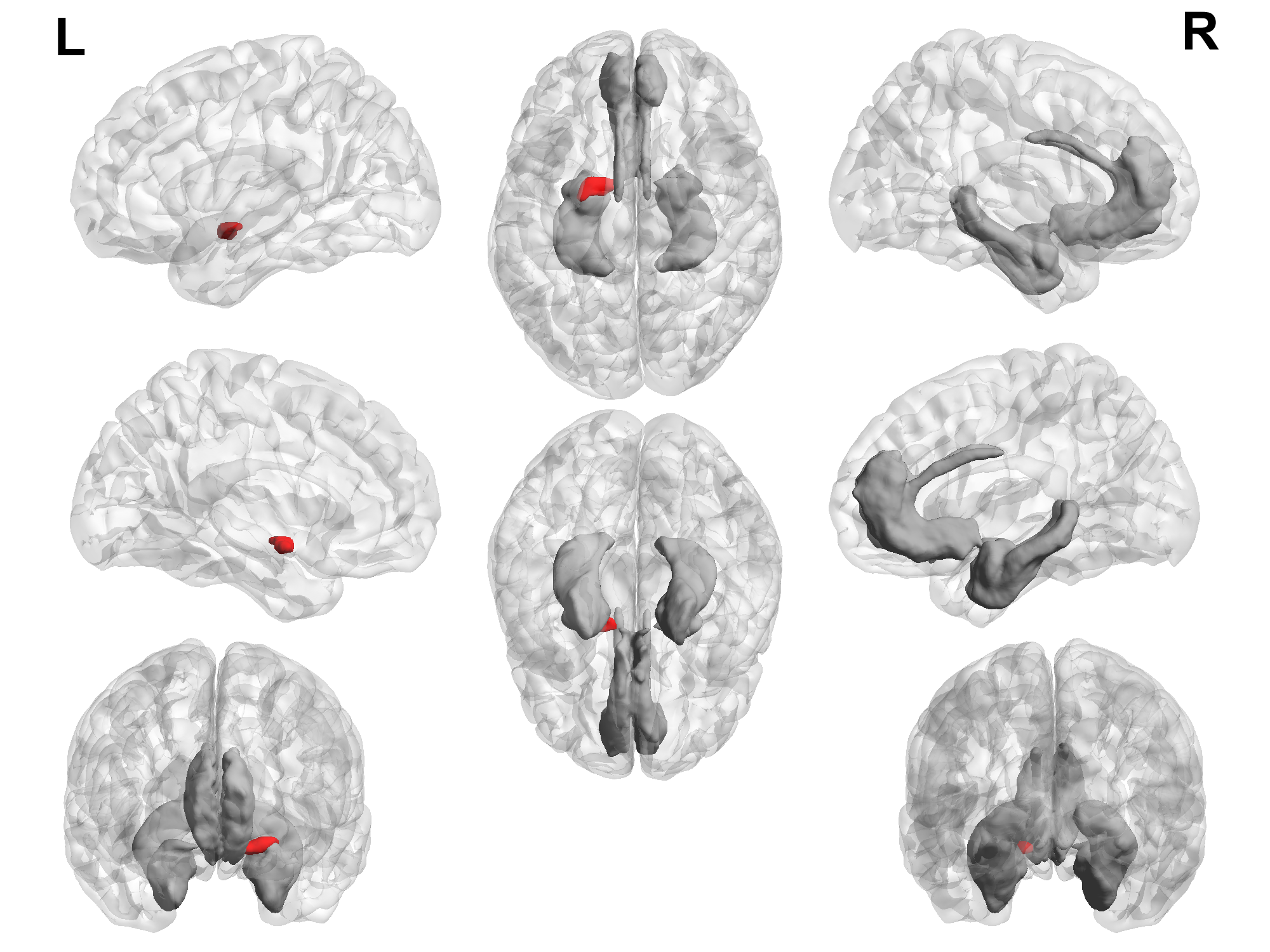


(12) CBF elevation in CI-nD group compared to NC group


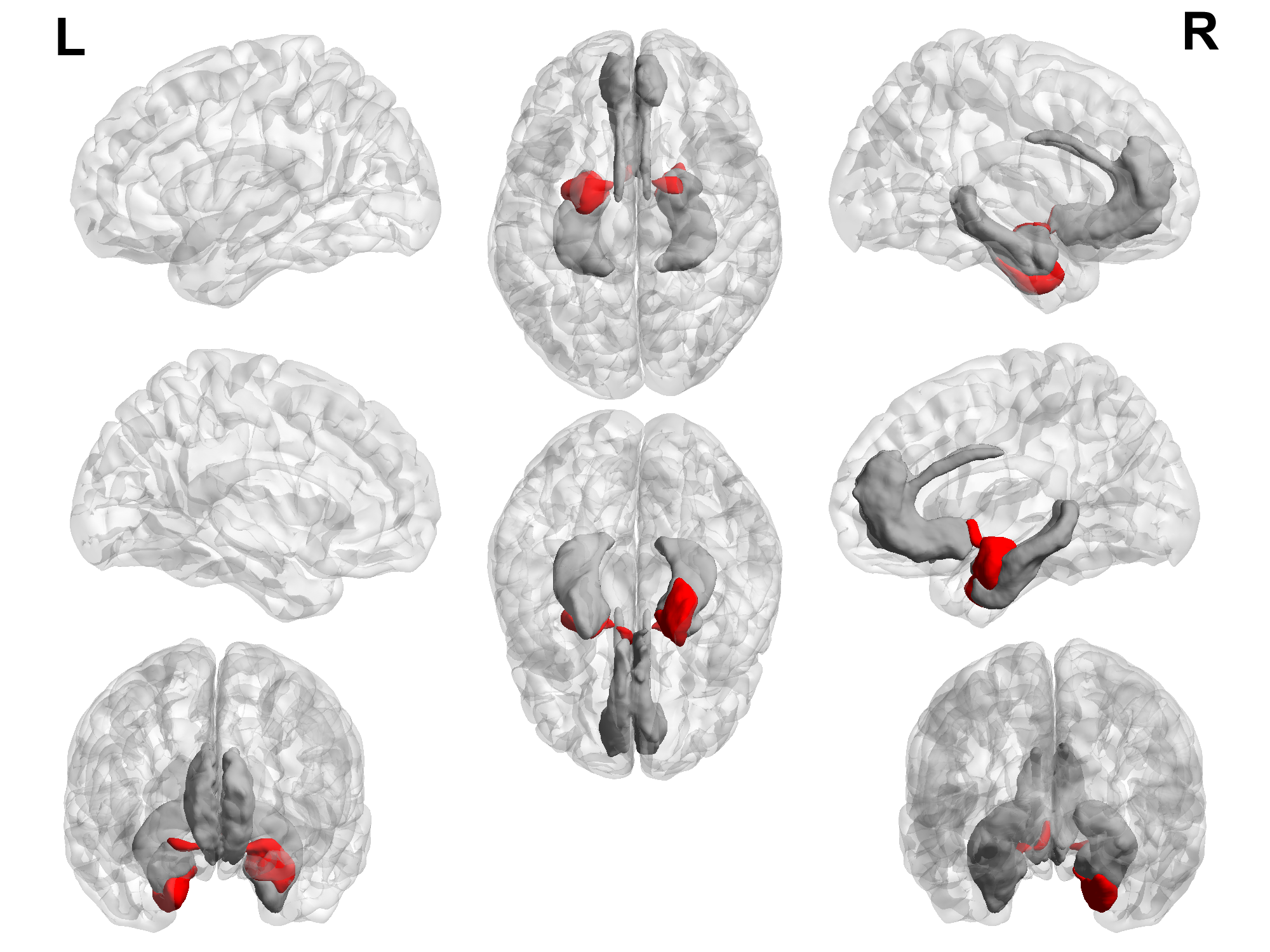


(13) CBF elevation in CI-D group compared to NC group


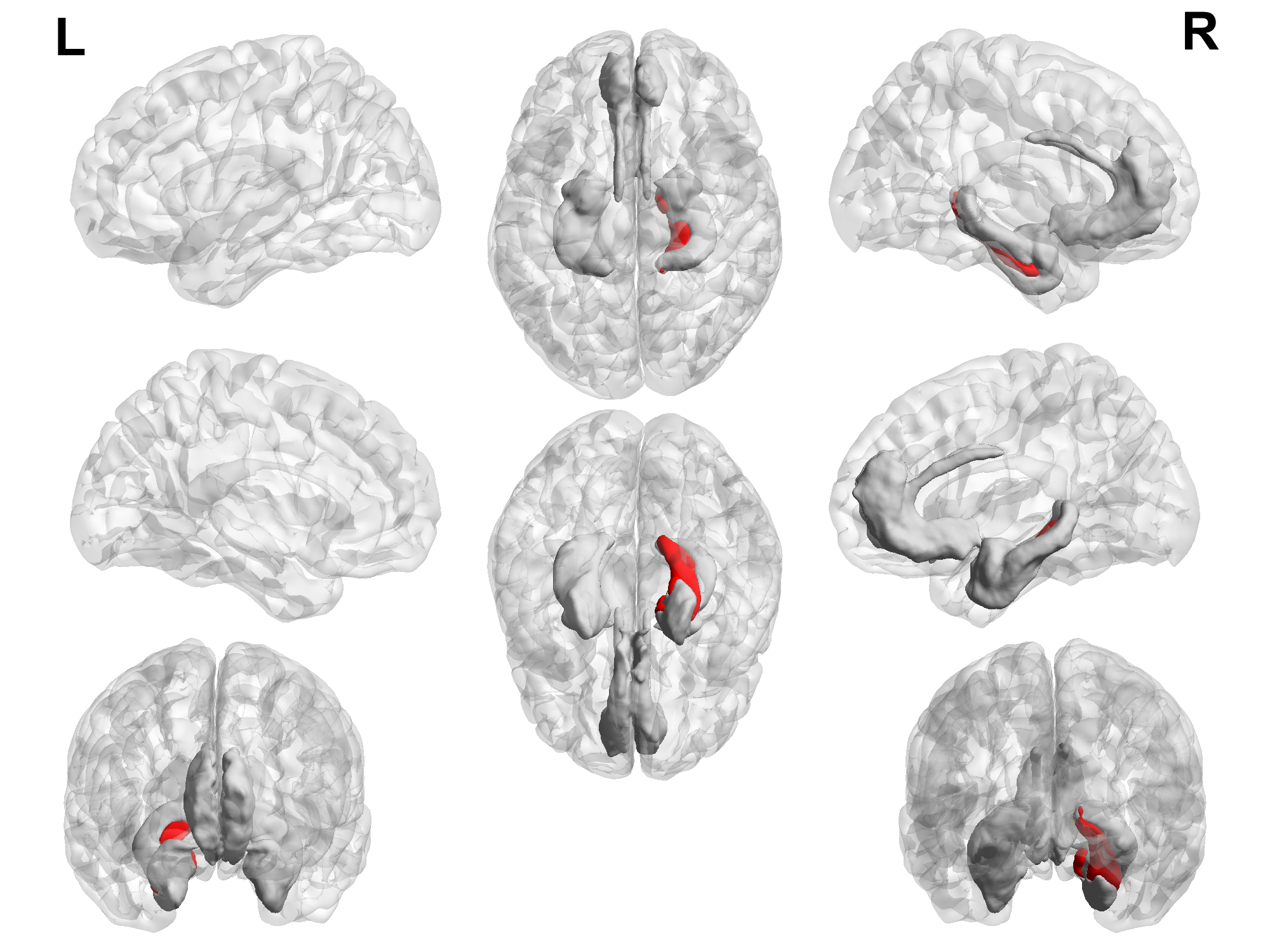
(14) CBF elevation in CI-D group compared to CI-nD group


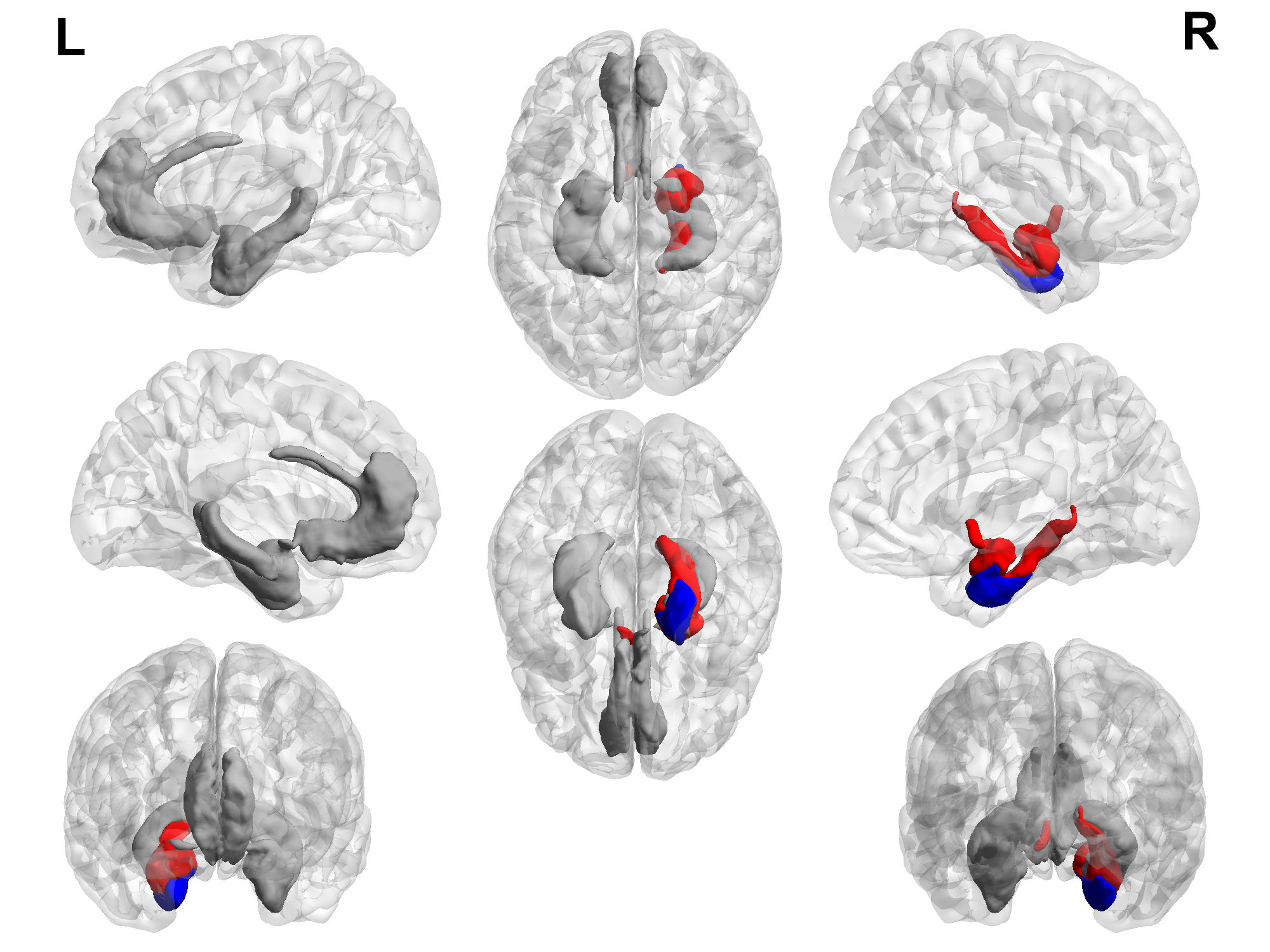


(15) QSM elevation and reduction in CI-nD group compared to NC group
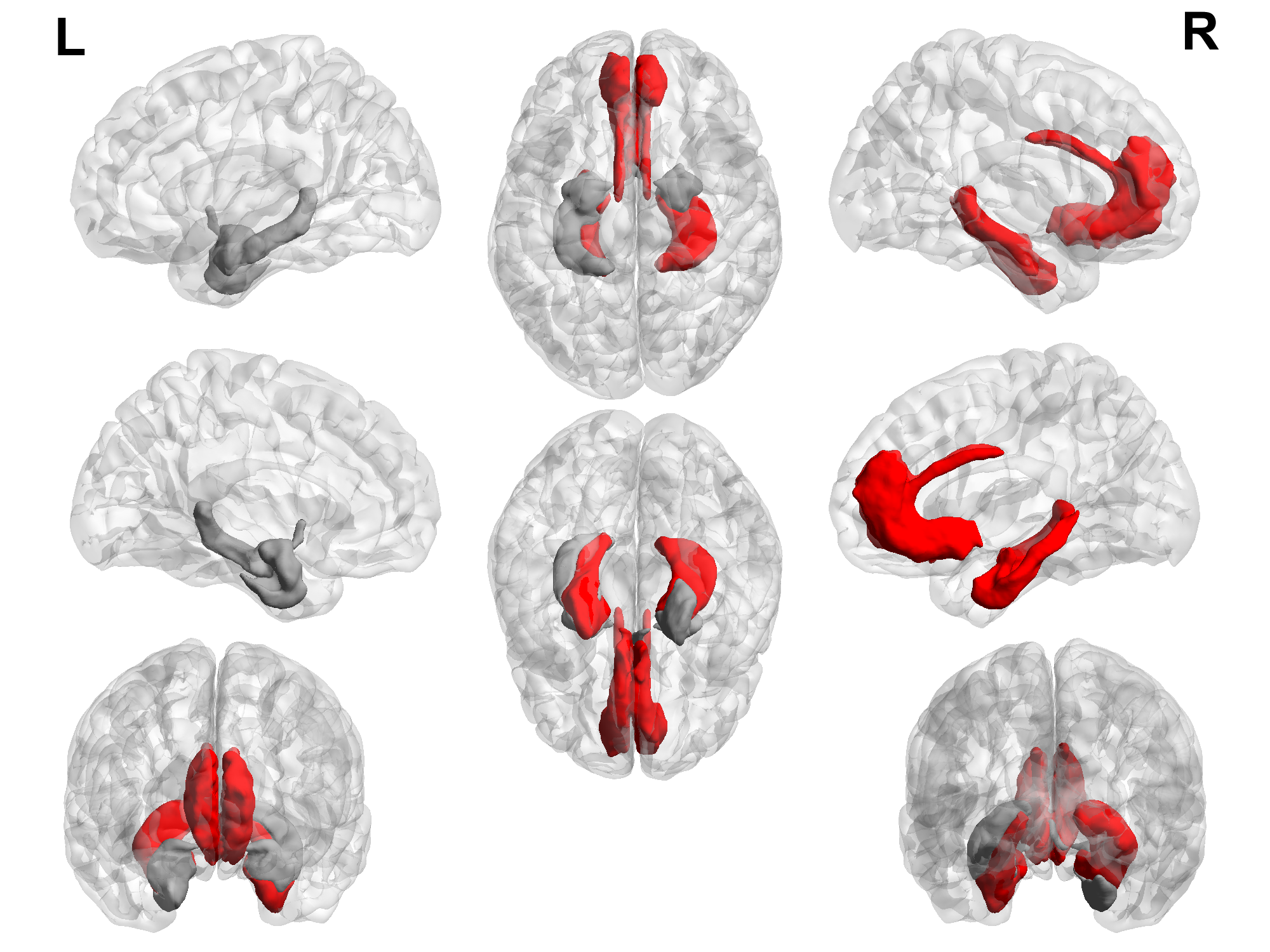
(16) QSM elevation in CI-D group compared to NC group


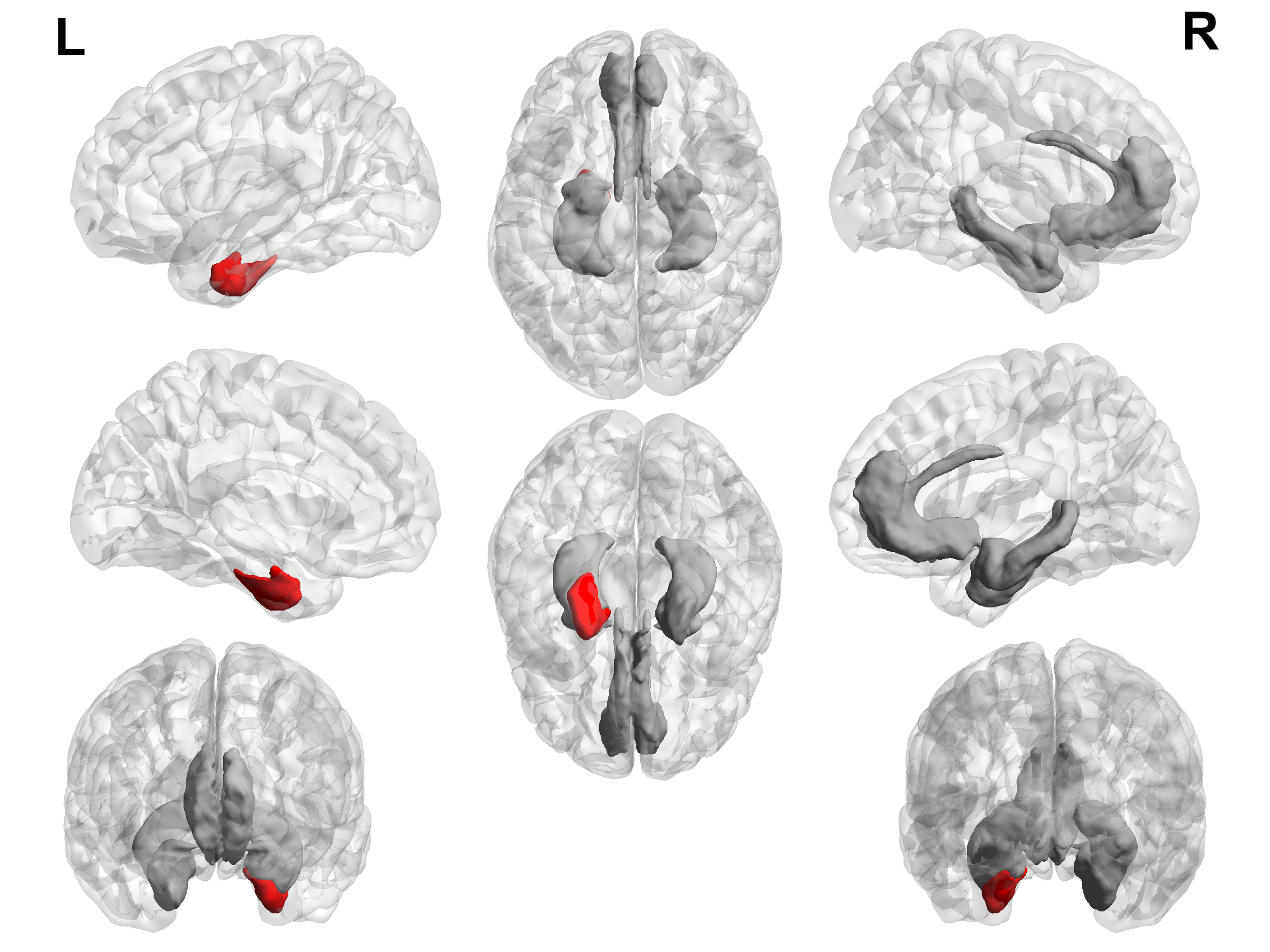
(17) QSM elevation in CI-D group compared to CI-nD group

FIGURE S3 Indirect effect of free water on cognitive function via VBM: Path analysis

Note:This figure illustrates the pathway analysis of two free water metrics (FW_CH123 and FW_CH4) on cognitive function (MoCA scores) through brain volume changes (VBM_CH123 and VBM_CH4), based on structural equation modeling.

**REFERENCES**

[1] Carlson JM, Fang L. Attentional bias to threat and gray matter volume morphology in high anxious individuals. Cogn Affect Behav Neurosci 2022;22:600–9. https://doi.org/10.3758/s13415-021-00968-9.

[2] Zang Y-F, He Y, Zhu C-Z, Cao Q-J, Sui M-Q, Liang M, et al. Altered baseline brain activity in children with ADHD revealed by resting-state functional MRI. Brain Dev 2007;29:83–91. https://doi.org/10.1016/j.braindev.2006.07.002.

[3] Li H-J, Xu Y, Zhang K-R, Hoptman MJ, Zuo X-N. Homotopic connectivity in drug-naïve, first-episode, early-onset schizophrenia. J Child Psychol Psychiatry 2015;56:432–43. https://doi.org/10.1111/jcpp.12307.

[4] Zhang Z, Liao M, Yao Z, Hu B, Xie Y, Zheng W, et al. Frequency-Specific Functional Connectivity Density as an Effective Biomarker for Adolescent Generalized Anxiety Disorder. Front Hum Neurosci 2017;11:549. https://doi.org/10.3389/fnhum.2017.00549.

[5] Liu D, Yan C, Ren J, Yao L, Kiviniemi VJ, Zang Y. Using coherence to measure regional homogeneity of resting-state FMRI signal. Front Syst Neurosci 2010;4:24. https://doi.org/10.3389/fnsys.2010.00024.

[6] Dumont M, Roy M, Jodoin P-M, Morency FC, Houde J-C, Xie Z, et al. Free Water in White Matter Differentiates MCI and AD From Control Subjects. Front Aging Neurosci 2019;11:270. https://doi.org/10.3389/fnagi.2019.00270.

[7] Guo W, Zhang D, Sun J, Chen L, Wu T, Xu E. Quantitative susceptibility mapping of subcortical iron deposition in Parkinson disease and multiple system atrophy: clinical correlations and diagnostic implications. Quant Imaging Med Surg 2024;14:4464–74. https://doi.org/10.21037/qims-24-168.

[8] Li W, Jiang J, Zou X, Zhang Y, Sun M, Jia Z, et al. The characteristics of arterial spin labeling cerebral blood flow in patients with subjective cognitive decline: The Chinese imaging, biomarkers, and lifestyle study. Front Neurosci 2022;16:961164. https://doi.org/10.3389/fnins.2022.961164.

[9] Kurth F, Gaser C, Luders E. A 12-step user guide for analyzing voxel-wise gray matter asymmetries in statistical parametric mapping (SPM). Nat Protoc 2015;10:293–304. https://doi.org/10.1038/nprot.2015.014.

[10] Yan C-G, Wang X-D, Zuo X-N, Zang Y-F. DPABI: Data Processing & Analysis for (Resting-State) Brain Imaging. Neuroinformatics 2016;14:339–51. https://doi.org/10.1007/s12021-016-9299-4.

[11] Tomasi D, Volkow ND. Functional connectivity density mapping. Proc Natl Acad Sci U S A 2010;107:9885–90. https://doi.org/10.1073/pnas.1001414107.

[12] Pasternak O, Westin C-F, Bouix S, Seidman LJ, Goldstein JM, Woo T-UW, et al. Excessive extracellular volume reveals a neurodegenerative pattern in schizophrenia onset. J Neurosci 2012;32:17365–72. https://doi.org/10.1523/JNEUROSCI.2904-12.2012.

[13] Pasternak O, Sochen N, Gur Y, Intrator N, Assaf Y. Free water elimination and mapping from diffusion MRI. Magn Reson Med 2009;62:717–30. https://doi.org/10.1002/mrm.22055.

[14] Maier-Hein KH, Neher PF, Houde J-C, Côté M-A, Garyfallidis E, Zhong J, et al. The challenge of mapping the human connectome based on diffusion tractography. Nat Commun 2017;8:1349. https://doi.org/10.1038/s41467-017-01285-x.

[15] Harada T, Kudo K, Fujima N, Yoshikawa M, Ikebe Y, Sato R, et al. Quantitative Susceptibility Mapping: Basic Methods and Clinical Applications. Radiographics 2022;42:1161–76. https://doi.org/10.1148/rg.210054.

[16] Wang Y, Liu T. Quantitative susceptibility mapping (QSM): Decoding MRI data for a tissue magnetic biomarker. Magn Reson Med 2015;73:82–101. https://doi.org/10.1002/mrm.25358.

[17] Alsop DC, Detre JA, Golay X, Günther M, Hendrikse J, Hernandez-Garcia L, et al. Recommended implementation of arterial spin-labeled perfusion MRI for clinical applications: A consensus of the ISMRM perfusion study group and the European consortium for ASL in dementia. Magn Reson Med 2015;73:102–16. https://doi.org/10.1002/mrm.25197.

[18] Hoshi A, Yamamoto T, Shimizu K, Ugawa Y, Nishizawa M, Takahashi H, et al. Characteristics of aquaporin expression surrounding senile plaques and cerebral amyloid angiopathy in Alzheimer disease. J Neuropathol Exp Neurol 2012;71:750–9. https://doi.org/10.1097/NEN.0b013e3182632566.

[19] Baldeiras I, Santana I, Leitão MJ, Gens H, Pascoal R, Tábuas-Pereira M, et al. Addition of the Aβ42/40 ratio to the cerebrospinal fluid biomarker profile increases the predictive value for underlying Alzheimer’s disease dementia in mild cognitive impairment. Alzheimers Res Ther 2018;10:33. https://doi.org/10.1186/s13195-018-0362-2.

[20] Kim K, Jang YJ, Shin J-H, Park MJ, Kim HS, Seong J-K, et al. Amyloid deposition and its association with depressive symptoms and cognitive functions in late-life depression: a longitudinal study using amyloid-β PET images and neuropsychological measurements. Alzheimers Res Ther 2024;16:232. https://doi.org/10.1186/s13195-024-01562-0.

[21] Thijssen EH, La Joie R, Strom A, Fonseca C, Iaccarino L, Wolf A, et al. Plasma phosphorylated tau 217 and phosphorylated tau 181 as biomarkers in Alzheimer’s disease and frontotemporal lobar degeneration: a retrospective diagnostic performance study. Lancet Neurol 2021;20:739–52. https://doi.org/10.1016/S1474-4422(21)00214-3.

[22] Ashton NJ, Leuzy A, Lim YM, Troakes C, Hortobágyi T, Höglund K, et al. Increased plasma neurofilament light chain concentration correlates with severity of post-mortem neurofibrillary tangle pathology and neurodegeneration. Acta Neuropathol Commun 2019;7:5. https://doi.org/10.1186/s40478-018-0649-3.

[23] Neurofilament Light Chain Is a Novel Biomarker for Major Depression and Related Executive Dysfunction - PubMed n.d. https://pubmed.ncbi.nlm.nih.gov/34637515/ (accessed January 26, 2025).
